# SC134-TCB Targeting Fucosyl-GM1, a T Cell–Engaging Antibody with Potent Antitumor Activity in Preclinical Small Cell Lung Cancer Models

**DOI:** 10.1158/1535-7163.MCT-24-0187

**Published:** 2024-08-26

**Authors:** Foram Dave, Poonam Vaghela, Bryony Heath, Zuzana Dunster, Elena Dubinina, Dhruma Thakker, Katie Mann, Joe Chadwick, Gaëlle Cane, Bubacarr G. Kaira, Omar J. Mohammed, Ruhul Choudhury, Samantha Paston, Tina Parsons, Mireille Vankemmelbeke, Lindy Durrant

**Affiliations:** 1 Scancell Ltd., Biodiscovery Institute, University of Nottingham, Nottingham, United Kingdom.; 2 Division of Cancer and Stem Cells, School of Medicine, University of Nottingham, Nottingham, United Kingdom.; 3 Scancell Ltd., Bellhouse Building, Oxford Science Park, Oxford, United Kingdom.

## Abstract

Small cell lung cancer (SCLC) is an aggressive disease with limited treatment options. Fucosyl-GM1 (FucGM1) is a glycolipid overexpressed in the majority of SCLC tumors but virtually absent from normal healthy tissues. In this study, we validate a FucGM1-targeting T cell–redirecting bispecific (TCB) antibody for the treatment of SCLC. More than 80% of patient-derived xenograft tissues of SCLC expressed FucGM1, whereas only three normal human tissues: pituitary, thymus, and skin expressed low and focal FucGM1. A FucGM1-targeting TCB (SC134-TCB), based on the Fc-silenced humanized SC134 antibody, exhibited nanomolar efficiency in FucGM1 glycolipid and SCLC cell surface binding. SC134-TCB showed potent *ex vivo* killing of SCLC cell lines with donor-dependent EC_50_ ranging from 7.2 pmol/L up to 211.0 pmol/L, effectively activating T cells, with picomolar efficiency, coinciding with target-dependent cytokine production such as IFNγ, IL2, and TNFα and robust proliferation of both CD4 and CD8 T cells. The *ex vivo* SC134-TCB tumor controlling activity translated into an effective *in vivo* anti-DMS79 tumor therapy, resulting in 100% tumor-free survival in a human peripheral blood mononuclear cell admixed setting and 40% overall survival (55% tumor growth inhibition) with systemically administered human peripheral blood mononuclear cells. Combination treatment with atezolizumab further enhanced survival and tumor growth inhibition (up to 73%). A 10-fold SC134-TCB dose reduction maintained the strong *in vivo* antitumor impact, translating into 70% overall survival (*P* < 0.0001). Whole-blood incubation with SC134-TCB, as well as healthy human primary cells analysis, revealed no target-independent cytokine production. SC134-TCB presents an attractive candidate to deliver an effective immunotherapy treatment option for patients with SCLC.

## Introduction

Small cell lung cancer (SCLC) is an aggressive neuroendocrine tumor associated with tobacco smoking, featuring rapid chemotherapy-resistant recurrence. Consequently, relapse rates are high, and 5-year survival rates are extremely low. Up to 15% of lung cancers fall within the SCLC subtype. First-line treatment for limited-stage SCLC (stages I–II), consists of surgery followed by chemotherapy, with or without radiotherapy (1). Extensive-stage (ES) SCLC (stages III and IV), often the stage at which patients present, has a much worse prognosis and is treated with a combination of platinum-based chemotherapy with or without immunotherapy (PDL1 inhibition). First-line PDL1 inhibition has somewhat improved outcomes [median overall survival (OS) improved by about 2 months], but patients still relapse; hence, there is an urgent need for more effective therapies.

T cell–redirecting bispecific (TCB) antibodies are a clinically validated treatment modality that are showing great promise, initially in hematologic cancers with CD19- or CD20-targeting TCB antibodies, but more recently also in solid tumors, in which encouraging response rates have been observed in uveal melanoma (targeting gp100) and multiple myeloma (targeting BCMA and GPRC5D; refs. 2, 3). Coengagement of tumor target and CD3 on T cells by TCB antibodies results in T-cell activation and target-dependent tumor cell killing, independent of MHC expression. This attractive mode of tumor killing necessitates the identification of tumor-specific targets to avoid off-tumor T cell–mediated toxicities.

Carbohydrate antigens are the most abundantly expressed cancer cell surface antigens (4). One such antigen is fucosyl-GM1 (FucGM1), a tumor-associated glycolipid. FucGM1 is composed of a ceramide lipid membrane component and a carbohydrate head group that is cell surface–exposed. It is expressed in 75% to 90% of SCLC cases (5) with little or no expression in normal tissues (6–12). The biosynthesis of FucGM1 involves several glycosyl transferases, some of which are overexpressed in SCLC, contributing to its progression (13–15). FucGM1 has been detected in culture media from SCLC cell lines, tumor extracts, serum of mouse xenografts, and in the serum of patients with ES-SCLC (16, 17). These reports provide convincing evidence for FucGM1 as a highly specific tumor antigen suitable for immunotherapeutic development. The SC134 mAb, generated through mixed cell and glycan conjugate immunizations, demonstrated specificity for FucGM1 and strong SCLC cell surface reactivity.

Here, we describe the first humanized anti–FucGM1 TCB, designed in an IgG-scFv “2 + 2” format, SC134-TCB. This was achieved by attaching the huOKT3-scFv to the carboxyl end of the humanized SC134 (h134) IgG1 light chain. The IgG1 backbone was further Fc-silenced to prevent Fc receptor–mediated adverse events. *In vitro*, SC134-TCB bound to FucGM1 and CD3 with nanomolar affinity. *Ex vivo*, SC134-TCB mediated picomolar target-dependent T-cell activation, proliferation, and potent SCLC cell killing. This culminated in significant *in vivo* DMS79 tumor control in NOD/SCIDγ (NSG) mice, resulting in 30% OS that further increased in combination with the PDL1 inhibitor atezolizumab. Critically, SC134-TCB presented little evidence for off-tumor T-cell activation. The tumor-selective nature of FucGM1 expression combined with the functional antitumor attributes of SC134-TCB suggests potential as a FucGM1-targeted TCB therapy for SCLC.

## Materials and Methods

### Creation of SC134 and engineering of the TCB

SC134 was created through classical hybridoma technology after BALB/c mice immunizations with DMS79 cells, followed by repeat FucGM1–HSA conjugates. Titers were assessed via ELISA for reactivity against purified FucGM1 (Matreya LLC) as well as for DMS79 cell surface binding. SC134 was obtained via limiting dilution cloning and isotyped as a murine IgG1 with the κ light chain. Following humanization of SC134 (h134), the SC134-TCB was created through fusion of the huOKT3-scFv (18) to the C-terminus of the light chain of h134, via a C-terminal (G4S)3 linker (19, 20). The Fc was silenced via introduction of three mutations at positions 234 to 236, the so-called “STR” methodology, recently shown to abolish all effector functions (21). Cloning, expression, and purification of the constructs were performed by evitria SA (details included in Supplementary Data).

### Cell lines and primary cell culture

SCLC cell lines, DMS79, DMS153, DMS53, H526, H146, and H740, were obtained and authenticated by ATCC and were cultured in RPMI 1640 (Sigma) or Waymouth MB 752/1 (Gibco) medium (DMS153) supplemented with 10% FBS (Sigma). FucGM1 expression levels were determined using the QIFIKIT (Agilent). The colorectal cell line COLO205 was purchased from ATCC and cultured in RPMI 1640 supplemented with 10% FBS. Cell lines were tested monthly for the presence of *Mycoplasma* and were used at low passage numbers for experiments. Human gastric fibroblast cells, obtained from ScienCell (#2830), were cultured in fibroblast medium (ScienCell, #2301). Human cardiac fibroblasts (#C-12375), human cardiac myocytes (#C-12810), normal human epithelial keratinocyte (#C-12013), normal human dermal fibroblasts (#C-12302), normal human epithelial melanocyte (#C-12413), and human aortic smooth muscle (#12533) were obtained from PromoCell. These were cultured in the respective medium recommended by the supplier. Human thymic fibroblasts (#P10490) and human thymic epithelial cells (#P10491) were purchased from Innoprot and cultured in fibroblast medium (Innoprot #P60108) and epithelial cell medium (Innoprot #P60108), respectively.

### IHC target expression analysis

Patient-derived tumor material was sourced from Crown Bioscience International and was acquired after approval by the IntegReview Ethical Review Board. The lipid nature of FucGM1 necessitated the use of frozen tissues for IHC target distribution analysis. The staining method was validated using smeared or optimal cutting temperature (OCT)–-embedded target-positive cells (DMS79). Frozen tissue sections were air-dried and rehydrated with cold PBS. The Human-on-Human kit (HOH-3000, VectorLabs) was used for staining human tissues with huIgG1 antibodies (h134 and isotype control; #31154, Thermo Fisher Scientific) according to the manufacturer’s protocol. Thyroid transcription factor 1 was also included as a marker for pulmonary small cell carcinoma (Supplementary Fig. S1E). Briefly, after blocking of endogenous peroxidase activity with hydrogen peroxide and a subsequent protein block, primary antibodies were applied using a kit-specific diluent mixture, followed by detection with horse radish peroxidase (HRPO)–linked anti–goat IgG polymer and DAB. Hematoxylin-modified Mayer’s solution was used for counterstaining. Slides were dehydrated, mounted, and scanned on NanoZoomer-SQ scanner. QuPath software V.3 was used for biomarker scoring. Summary scores were calculated, and each tissue core or section was assigned a H-score ranging from 0 to 300, based on staining intensity and percentage positive cells.

### Effector cell preparation

Human peripheral blood mononuclear cells (huPBMC) were obtained from Cambridge Bioscience or isolated from healthy donors or leukopak (BioIVT) through density gradient centrifugation. Pan–T cells were enriched using the Pan T Cell Immunoaffinity Isolation Kit (Miltenyi Biotec). All donors provided written informed consent.

### TCB functional binding assays

TCB target binding was analyzed by flow cytometry and ELISA. For flow cytometry, 1 × 10^5^ effector or SCLC cells were incubated with serially diluted TCB antibodies for 1 hour at 4°C. Following washing, bound TCB antibodies was detected using FITC-labeled anti–human IgG Fc (Sigma). Stained samples were washed and resuspended in Intracellular Fixation buffer (Invitrogen), followed by analysis on a MACSQuant 16 flow cytometer, equipped with MACSQuant software version 2.13.3, using secondary antibody–stained controls to determine suitable gates. The ability of TCB antibodies to bind CD3ED (ACROBiosystems Inc.) and FucGM1 lipid (Matreya LLC) was determined by direct ELISA. Briefly, high-binding ELISA plates were coated with 200 ng/well FucGM1 or CD3ED, followed by blocking with protein-free blocking buffer (Thermo Fisher Scientific). Primary TCB dilutions were detected using anti–human IgG (γ-chain specific)–biotin antibody (Sigma), followed by a streptavidin–HRPO conjugate.

### Cell-based functional assays

Effector cells (1 × 10^5^, huPBMCs or pan–T cells) were cocultured with target cells and serially diluted TCB for 48 hours, at an effector to target ratio (E:T) of 5:1, after which two complementary cytotoxicity and activation assays were performed. Lactate dehydrogenase (LDH) release in the culture supernatant was measured using the CytoTox 96 Non-Radioactive Cytotoxicity Assay (Promega), in which maximum lysis was established by using the kit-specific cell lysis buffer. The percentage cytotoxicity was calculated using the formula (experimental – effector spontaneous – target spontaneous)/(target maximum – target spontaneous) × 100, and the target and effector cells were further analyzed by flow cytometry. The percentage of dead target cells was determined using anti-EpCAM-Alexa Fluor 647 (BioLegend) to enable target cell gating, in combination with Live/Dead Yellow stain (MACSQuant 16). Additionally, T-cell activation was determined using labeled antibodies directed to CD69, CD4, and CD8 (BioLegend). T-cell proliferation was analyzed using intracellular staining of Ki67 using directly labeled antibody (Invitrogen #12-5698-82). In some assays, catumaxomab, an EpCAM-targeting TCB was included (MedChemtronica AB, HY-P9954).

Effector cell activation after overnight incubation in the presence of SC134-TCB (E:T, 5:1 or 2:1 for primary cells) was also analyzed through ELISpot assays (ELISpot Flex) using human IFNγ (ALP) capture and detection reagents according to the manufacturer’s instructions (Mabtech). Spots were analyzed using an automated plate reader (Cellular Technologies).

### Proinflammatory cytokine detection

Proinflammatory cytokines released in coculture medium or whole blood were detected using the Meso Scale Discovery platform (MSD) in combination with the human proinflammatory panel 1 kit. For TCB-induced cytokine detection in whole blood, serially diluted SC134-TCB and huOKT3 (10 nmol/L) positive control were incubated in whole blood of four healthy human donors for 5 or 24 hours at 37°C and 5% CO_2_. Following incubation, the plate was spun at 1,000 *g* for 10 minutes and serum harvested. Sera taken from whole blood assays and *in vivo* studies were diluted 1:2 in assay buffer, and the cytokine detection was performed according to the manufacturer’s protocol (MSD). Results were analyzed using Methodical Mind version 1.2.3 and MSD Discovery Workbench software version LSR_4_0_13.

### *In vivo* models

Female NSG mice (Charles River Laboratories), ages between 8 to 20 weeks old, were used for *in vivo* studies. Animal experiments were carried out with ethical approval from the University of Nottingham and Nottingham Trent University and under a Home Office–approved project license. Mice were randomized into treatment groups, not blinded to the investigators. For admixing experiments, 2 × 10^6^ DMS79 and huPBMCs were premixed at a ratio of 1:1 and inoculated subcutaneously in the right flank in 2.5:1 PBS and Matrigel (Corning Life Sciences), and PBMC (2 × 10^6^) were intravenously re-administered on days 8 and 15. SC134-TCB (100 μg) was dosed intraperitoneally for three consecutive days starting on day 2, and this cycle was repeated on days 9 and 16.

In studies in which tumor and PBMCs were dosed separately, mice were inoculated (subcutaneously) in the right flank, on day 1 with 2 × 10^6^ DMS79 cells, in 1:1 PBS and Matrigel. huPBMCs were administered (iv) on days 3 and 18. SC134-TCB was dosed (100 μg, i.p.) biweekly for 3 weeks, starting on day 4. A tail bleed was taken 24 hours after the initial SC134-TCB dose. Tumor growth was monitored biweekly, and following euthanasia, the terminal blood, tumor, and spleen were taken for further analysis.

### Terminal sample analysis *in vivo* studies

For tumor-infiltrated T cells (Tils) analysis, tumors were disaggregated and effector cells were labeled with CD4 efluor 450 (RPA-T4) and CD8 efluor 450 (RPA-T8; Miltenyi Biotec), anti–PD1 APC-Cy7 (BioLegend), and anti–TIM3 PE-Cy7 (BioLegend). For FucGM1 expression, tumor cells were labeled with anti–EpCAM AFG47 (BioLegend) and h134 plus antihuman IgG FITC secondary antibodies (Sigma). Spleens were harvested, and splenocytes were isolated and stained with relevant T-cell antibodies, as above. The presence of TCB was evaluated (tumor and spleen) using an anti–human IgG (Fc-specific) FITC secondary antibody (Sigma). Sections from formalin-fixed, paraffin-embedded–processed tumor samples were stained for the presence of CD4 and CD8 T cells using rabbit anti–human CD8 (05937248001, Roche) and rabbit anti–human CD4 (AB213215, Abcam) and for tumur cells using rabbit anti–human thyroid transcription factor 1 (Abcam, ab133737) and rabbit isotype control (172730, Abcam).

### *In vivo* antibody pharmacokinetics

Female NSG mice (Charles River Laboratories) were injected with 100 µg of SC134-TCB and serially bled over 1 to 144 hours. Sandwich ELISA was used to determine TCB serum concentration. Briefly, wells were coated with anti–human IgG capture antibody (Sigma), followed by blocking and incubation with serum samples diluted 1:100 and 1:1,000. Captured TCB was detected via incubation with anti–light chain HRPO-labeled antibody (Sigma). Following incubation, plates were developed with TMB Core+ (Bio-Rad) and read at 490 nm using Tecan Infinite M Plex plate reader. The absorbance of known SC134-TCB concentrations was used to generate the standard curve against which measured values were interpolated (GraphPad Prism software V.10).

### Statistical analysis

Statistical analyses were carried out using GraphPad Prism software V.10. For all statistical tests, a *P* value of <0.05 was used to ascribe statistical significance. All error bars denote the SD, unless otherwise noted.

### Data availability

Raw data for this study were generated at Scancell Ltd. Derived data supporting the findings of this study are available from the corresponding author upon request.

## Results

### SC134 obtained via mixed SCLC cell (DMS79) and FucGM1–HSA immunizations specifically targets FucGM1 lipid

**Figure 1.**
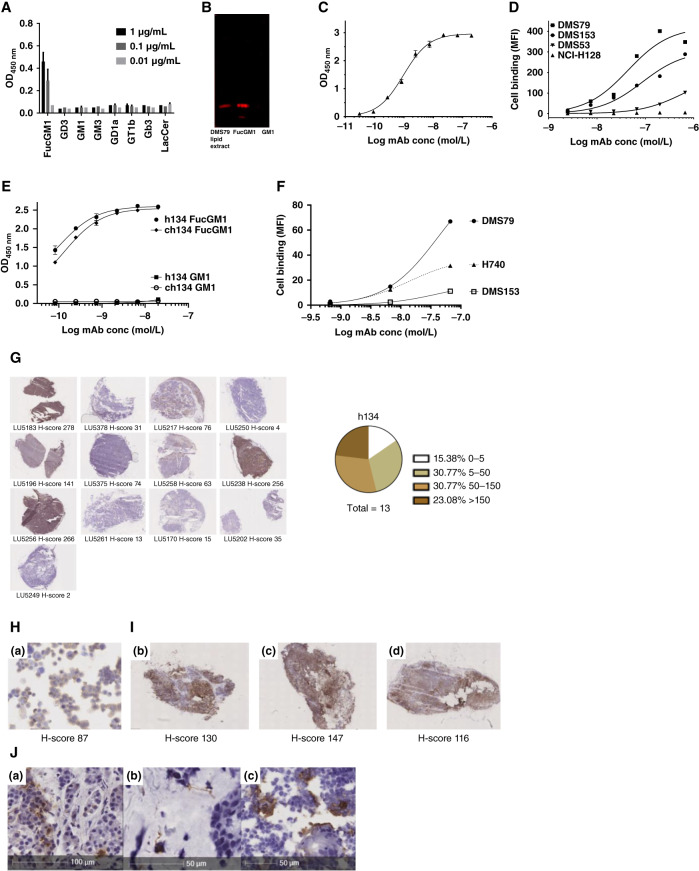
SC134 specifically binds FucGM1, a SCLC selective target. Glycolipid binding specificity by hybridoma-derived SC134. **A,** ELISA analysis of FucGM1-binding specificity of SC134. FucGM1 binding is observed, in contrast to lack of binding to closely related gangliosides. Positive control antibody reactivity for the respective gangliosides is shown in Supplementary Fig. S1A. **B,** Immuno-TLC analysis of SC134 showing reactivity toward DMS79 lipid extract as well as purified FucGM1, combined with lack of GM1 reactivity. **C,** SC134-binding efficiency for FucGM1 on ELISA. **D,** SC134 cell surface–binding efficiency on a range of SCLC cell lines. h134 characterization: (**E**) FucGM1 lipid binding compared with GM1 by ch134 and h134 on ELISA. **F,** SCLC cell line binding by h134. MFI assessed by flow cytometry. FucGM1 distribution in SCLC tumor tissues: (**G**) IHC FucGM1 expression analysis using h134 (huIgG1) of frozen patient-derived xenograft SCLC tissues. **H,** FucGM1 expression by DMS79 in culture. **I,** FucGM1 expression by DMS79 after growing in NSG mice. **J,** Restricted FucGM1 expression in frozen normal healthy human tissues, immunostained with h134 (**J**). The pituitary (a), skin (b), and thymus (c) were the only positive tissues from the 30 normal healthy tissues analyzed. The full arrays are shown in Supplementary Fig. S1D. ch134, chimeric (human IgG1) SC134; MFI, median fluorescence intensity; TLC, thin-layer chromatography.

Hybridoma-derived SC134 is a murine IgG1 isotype with the κ light chain, with variable regions belonging to mouse heavy-chain IGHV3-1*02 F (IGHJ4*01 F and IGHD5-1*01 P) and light-chain IGKV5-39*01 (FIGKJ1*01 F). FucGM1 binding specificity of SC134 was ascertained via glycolipid ELISA across a panel of commercially available glycolipids (Fig. 1A; Supplementary Fig. S1A) as well as using thin-layer chromatography of DMS79 glycolipid extract and commercially purified FucGM1, with detection by immunoblotting using SC134 (Fig. 1B). Across both techniques, SC134 specifically targeted FucGM1 with no cross-reactivity to GM1, nor to closely related gangliosides. SC134 bound FucGM1 with nanomolar efficiency on ELISA (Fig. 1C), as well as for cell surface binding, across a range of SCLC cell lines, including DMS79, DMS153, and DMS53 (Fig. 1D). Additionally, preincubation of SC134 with FucGM1 blocked DMS79 cell surface binding (Supplementary Fig. S1B), further supporting the binding specificity of SC134. Expression levels of FucGM1 in a subset of SCLC cell lines ranged from ∼500,000 specific antibody–binding capacity for DMS79 and DMS153 to ∼100,000 for DMS53 (Supplementary Fig. S1C).

### FucGM1 is an effective SCLC target with limited normal tissue expression

In order to facilitate clinical translation, a humanized lead candidate, h134, a human IgG1 based on SC134, was created. h134, similarly to parental chimeric SC134 bound FucGM1 with an EC_50_ of 0.07 nmol/L, with no reactivity toward GM1, thus maintaining FucGM1 specificity (Fig. 1E). Crucially, h134 bound a range of SCLC cell lines, with DMS79 showing the highest binding levels, followed by H740 and DMS153 (Fig. 1F) and with EC_50_ values ranging from 14 nmol/L (H740) to 41 nmol/L (DMS79) and 42 nmol/L (DMS153). SCLC is rarely resected, and consequently, we relied on frozen patient-derived xenograft tissues of SCLC (Crown Bioscience UK), in which immunostaining with h134 was used to evaluate tumor FucGM1 expression. FucGM1 expression was evident in ∼85% (11/13) of the cases with 23% strong, 31% moderate, and 31% weak staining observed, with >60% of neoplastic cells staining positive for FucGM1 in the positive tissues (Fig. 1G). Laboratory-grown DMS79 (Fig. 1H; H-score of 87) and *in vivo* grown DMS79 xenograft tissues (Fig. 1I; H-scores of 131 ± 16) approximated or matched the H-scores of a large fraction of the patient-derived xenograft tissues, indicating that DMS79 is a valid model for SC134-TCB efficacy evaluation. In sharp contrast, across a FDA-approved multihealthy human tissue array, only three tissues exhibited isolated focal staining in a donor-dependent manner: pituitary (1/3), skin (2/3), and thymus (3/3; Fig. 1J), confirming the tumor specificity of FucGM1 expression. This prevalent SCLC tumor FucGM1 expression, together with its absence in most normal tissues (Supplementary Fig. S1D), supported the advancement of SC134 as a bispecific antibody for T-cell redirection.

### Nanomolar target binding by SC134-TCB

**Figure 2.**
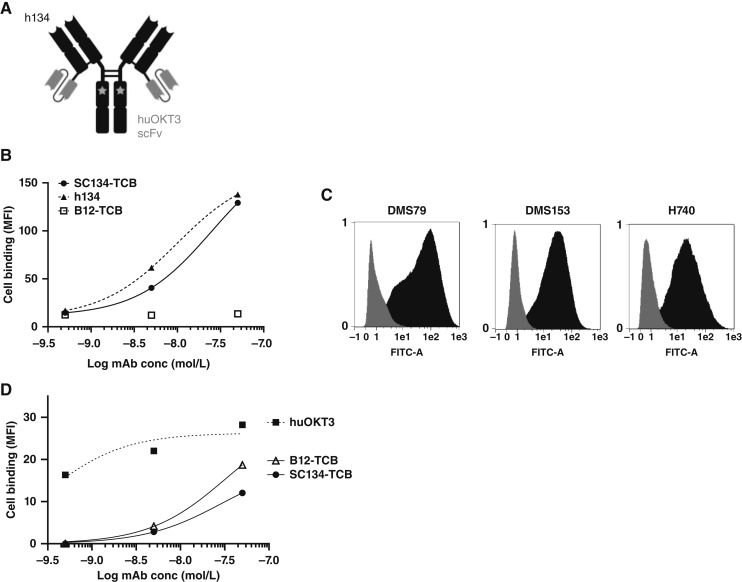
SC134-TCB effectively binds SCLC cell lines and T cells. **A,** Schematic drawing of the SC134-TCB format. Targeting regions of h134 in combination with scFv of huOKT3 were used. Stars denote “short tandem repeat” modification for Fc silencing. SCLC cell binding by SC134-TCB: (**B**) DMS79 cell binding of SC134-TCB and parental h134. **C,** Histograms of top concentration (50 nmol/L) SC134-TCB binding (black) to three target-positive cell lines DMS79, DMS153, and H740 compared with secondary antibody–only control (gray). **D,** Pan–T-cell binding of B12-TCB, SC134-TCB, and parental anti-CD3 (huOKT3). MFI assessed via flow cytometry. MFI, median fluorescence intensity. (**A,** Created with BioRender.com.)

SC134-TCB was created on the basis of a native human IgG1 antibody with the CD3-targeting scFv fused via a flexible linker to the light chain, thus enabling bivalent FucGM1 binding (Fig. 2A). Additionally, the Fc was rendered nonfunctional via the introduction of the “short tandem repeat” mutations at positions 234 to 236 that abolish all effector functions (21). In cell-based assays, SC134-TCB, like h134, exhibited avid DMS79 cell surface binding with an EC_50_ of 25.1 and 9.7 nmol/L, respectively, whereas the nontargeting B12-TCB did not show any DMS79 binding (Fig. 2B; Supplementary Table S1). FucGM1 binding was also observed on DMS153 and H740, with an EC_50_ of 33.9 and 17.8 nmol/L, respectively (Fig. 2,C; Supplementary Table S1). No SC134-TCB binding was observed on target-negative cell lines (Supplementary Fig. S2). The CD3-targeting scFv was derived from the humanized OKT3 sequence (18) and fused to the C-terminus of the h134 light chain through a 15-residue flexible linker, an approach that previously has been shown to render the anti-CD3 portion “functionally monovalent” (19). This was evident from the pan–T-cell binding in which the parental huOKT3 bound T cells with 0.3 nmol/L EC_50_, compared with the EC_50_ of 29.2 and 32.5 nmol/L for SC134-TCB and B12-TCB, respectively (Fig. 2D; Supplementary Table S1).

### SC134-TCB exhibits potent target-dependent T cell–mediated SCLC killing

**Figure 3.**
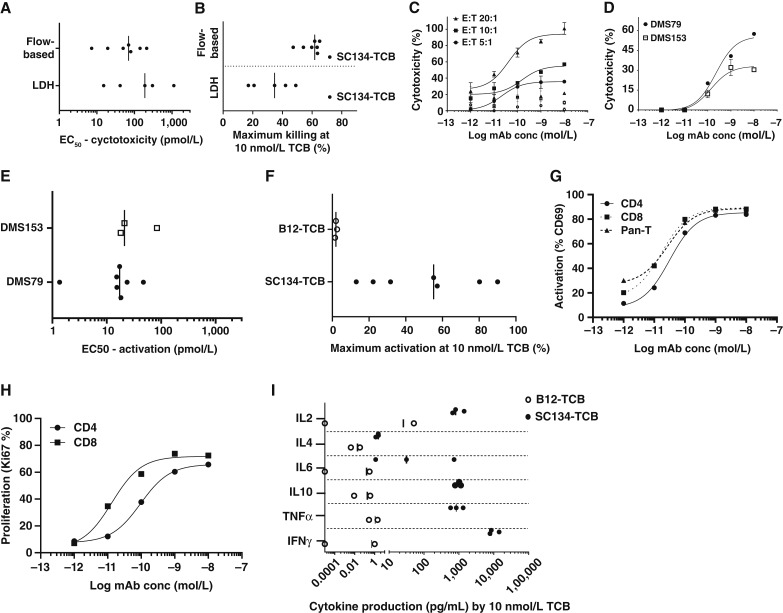
Potent T cell–mediated tumor cell killing by SC134-TCB. SC134-TCB induced tumor cell killing. **A,** Cytotoxicity EC_50_ via LDH release and flow-based analysis in pan-T:DMS79 cocultures. **B,** Percentage maximum target cell death of DMS79 with SC134-TCB and isotype control B12-TCB at 10 nmol/L. **C,** Increased killing by SC134-TCB (LDH release) at higher E:T ratios. Open symbols denote background killing by isotype control B12-TCB. **D,** LDH release from two target cell lines: high-expressor DMS79 and moderate-expressor DMS153. SC134-TCB induced T-cell activation. **E,** EC_50_ via CD69 staining shown for DMS79 and DMS153. **F,** Percentage maximum activation with SC134-TCB and isotype control B12-TCB at 10 nmol/L. **G,** Activation of CD4 and CD8 T-cell subsets from one effector donor. **H,** SC134-TCB induced CD4 and CD8 T-cell proliferation (day 3) determined by Ki67 staining. Representative donor shown. **I,** Target-dependent multifunctional cytokine production by SC134-TCB in DMS79 cocultures with enriched T cells. T cells used at an E:T of 5:1, for all assays. Median or representative data shown for three or more human donors.

SC134-TCB induced T cell–redirected lysis in a donor-dependent manner with subnanomolar to picomolar efficiency, using pan–T cells as effectors and DMS79 as target at an E:T ratio of 5:1. The median EC_50_ was 190 and 70 pmol/L for LDH and flow-based killing, respectively (Fig. 3A). The percentage maximum killing varied depending on the donor and assay used, with up to 50% (LDH) and 60% (flow-based killing) observed (Fig. 3B). Increasing the E:T ratio further increased maximum killing up to 100% (LDH release, Fig. 3C), coinciding with a slightly raised background from the nontargeting control B12-TCB. Additionally, SC134-TCB demonstrated superior killing efficiency compared with an EpCAM-targeting control TCB (Supplementary Fig. S3C). Overall, both assays showed good concordance, with flow-based killing being more sensitive than LDH release. SC134-TCB–mediated SCLC killing was target-dependent as the nontargeting control B12-TCB showed no killing using LDH release or much reduced, donor-dependent killing (flow-based analysis), likely reflecting the more sensitive nature of the latter assay (Supplementary Fig. S3A). Additionally, SC134-TCB exhibited no killing of the target-negative H146 cell line (Supplementary Fig. S3B). SC134-TCB also induced killing on the more moderately FucGM1-expressing DMS153, with comparable efficiency but reduced maximal killing (Fig. 3D). SC134-TCB–mediated cytotoxicity by pan–T cell and huPBMC was largely matched, with subtle differences depending on the assay (Supplementary Fig. S4).

### SC134-TCB mediates potent target-dependent T-cell activation, proliferation, and multifunctional cytokine production

SC134-TCB exhibited picomolar T-cell activation (based on CD69 expression) in a donor-dependent manner on coincubation with DMS79 or DMS53 (Fig. 3E) with maximum activation ranging from 20% to nearly 90% for coincubation with DMS79. T-cell activation was strictly target-dependent as no activation was evident with the nontargeting B12-TCB (Fig. 3F) nor in cocultures with target-negative human umbilical vein endothelial cells (Supplementary Fig. S5). Activation of both CD4 and CD8 T cells was achieved with picomolar efficiency (Fig. 3G). Target-dependent T-cell proliferation, based on Ki67 staining, was also evident for both CD4 and CD8 T cells, with picomolar efficiency over a 3-day coincubation with DMS79, with EC_50_ values for CD8 T-cell proliferation being lower than that of CD4 T cells, at 13.4 and 95.7 pmol/L, respectively (Fig. 3H). SC134-TCB–mediated T-cell activation triggered multifunctional cytokine production that was comparable between pan–T cells and huPBMCs (Fig. 3I; Supplementary Fig. S6A). IFNγ was the dominant cytokine across both effector types and was produced with picomolar efficiency (Supplementary Fig. S6B). Cytokine production by the nontargeting B12-TCB remained low in comparison, indicating the strict target dependency of the T-cell activation (Supplementary Fig. S6B).

### SC134-TCB exerts effective *in vivo* tumor control

**Figure 4.**
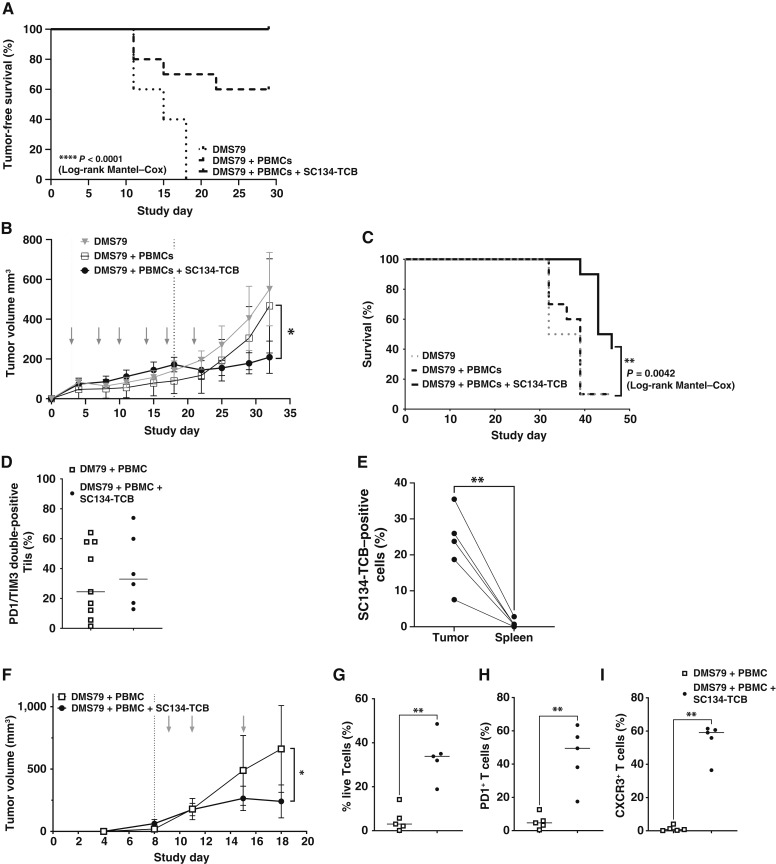
Effective tumor control by SC134-TCB. **A,** Significant tumor-free survival in admixed antitumor study dosed with 100 μg SC134-TCB for 3 weeks. PBMC implanted with DMS79 cells at 1:1 ratio and a further two doses of PBMCs given on days 8 and 15. Log-rank Mantel–Cox *P* < 0.0001. Significant DMS79 tumor control by SC134-TCB treatment, with PBMCs dosed on days 3 and 18 (dotted lines), after tumor inoculation, at 5:1 ratio. Gray arrows show biweekly dosing of SC134-TCB (100 μg): (**B**) tumor volume (individual growth curves, shown in Supplementary Fig. S9A). Significance, mixed-effects model *P* = 0.013 and (**C**) OS, log-rank Mantel–Cox *P* = 0.0042, shown. **D,** Phenotyping data assessing the percentage of exhausted T cells (PD1^+^TIM3^+^) in terminal tumor samples. T cells were gated based on combined CD4 and CD8 staining (gating strategy depicted in Supplementary Fig. S9B). **E,** SC134-TCB detection in tumors compared with spleens in the SC134-TCB–dosed group (i.v.), paired *t* test, *P* = 0.0069. Each point represents an individual mouse. SC134-TCB–mediated T-cell infiltration and activation coincide with significant antitumor impact. **F,** Significant tumor control by SC134 with single PBMC administration (day 8) combined with SC134-TCB (100 μg at days 9, 11, and 15). Significance, two-way ANOVA, *P* = 0.0249; individual growth curves are shown in Supplementary Fig. S9C. **G,** Significantly increased T-cell presence and T-cell activation: (**H**) PD1^+^ and (**I**) CXCR3^+^ in tumors of SC134-TCB–treated mice. The presence of individual populations is evident from the IHC analysis of terminal tumor samples (Supplementary Fig. S9D).

The efficient SC134-TCB *in vitro* SCLC cell killing encouraged us to validate its activity on *in vivo* tumor growth. The dosing schedule was selected on the basis of the observed plasma clearance profile in a single-dose study (100 μg, i.v.) in non–tumor-bearing NSG mice, in which plasma concentration of SC134-TCB remained stable for up to 6 days (Supplementary Fig. S7). The *in vivo* impact of SC134-TCB was first tested in NSG mice inoculated with admixed huPBMCs and DMS79 cells at a 1:1 ratio. In this setting, effector cells alone had a significant impact on DMS79 growth (Supplementary Fig. S8), resulting in 60% tumor-free survival (Fig. 4A). Biweekly administration of SC134-TCB significantly improved this outcome, resulting in 100% tumor-free survival. Importantly, SC134-TCB maintained its antitumor effect when huPBMCs were administered, after inoculation of tumor cells, on day 3. Biweekly administration of SC134-TCB in this setting led to a 45% (*P* = 0.013) tumor growth inhibition compared with the tumor plus effector control group (Fig. 4B), including one strongly regressing tumor by day 36 (Supplementary Fig. S9A). This coincided with a significant improvement in OS (*P* = 0.0042) in the SC134-TCB–treated group in which 40% OS was seen compared with 10% in the control group (Fig. 4C). Terminal tumor analysis revealed the presence of tumor-infiltrated T cells (Tils) in both the control and the SC134-TCB–treated group, ranging from 0.4% to 3.3% in the control group and 0.2% to 11.9% in the SC134-TCB–treated group. Increased levels of exhaustion, based on PD1 and Tim3 coexpression, were apparent in the SC134-TCB treatment group compared with the control group; this however, did not reach significance (Fig. 4D). Critically, in the SC134-TCB–treated mice, significantly more SC134-TCB could be detected in the terminal tumor samples compared with the near absence of SC134-TCB in the spleens (Fig. 4E), indicating that the majority of dosed SC134-TCB successfully targets the tumors. A condensed regimen follow-up study was performed with a single administration of huPBMCs and earlier tumor harvest, 3 days after the last TCB dose, when tumors showed active regression, to capture the mode of action of SC134-TCB. SC134-TCB led to a 64% tumor growth inhibition, *P* = 0.0249 (Fig. 4F), concomitant with a significantly increased CD4^+^ as well as CD8^+^ T-cell content (Fig. 4E; Supplementary Fig. S9D). The infiltrated T cells showed an activated, effector phenotype (PD1^+^ and CXCR3^+^, respectively; Fig. 4H and I).

### Combination with PDL1 inhibition (atezolizumab), as well as dose modulation, further improves SC134-TCB *in vivo* tumor control

**Figure 5.**
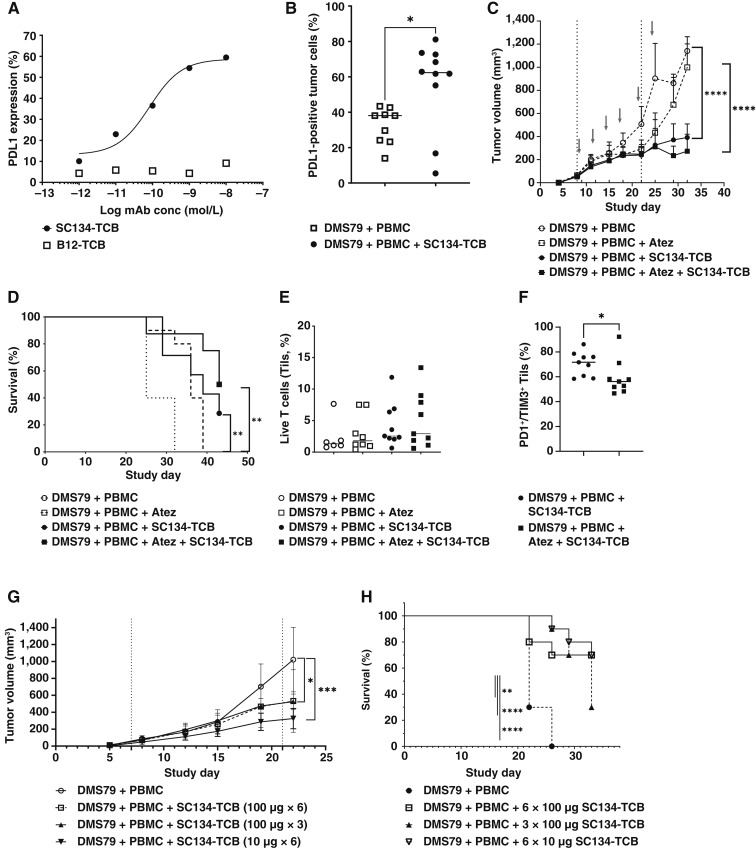
PDL1 inhibition and dose modulation further improve SC134-TCB antitumor impact. SC134-TCB treatment induces PDL1 expression. **A,** Dose-dependent PDL1 expression in *ex vivo* 48-hour cocultures (DMS79: huPBMCs at 5:1). **B,***In vivo* SC134-TCB–induced PDL1 expression on DMS79 cells (at termination) from NSG antitumor study, Mann–Whitney test, *P* = 0.0220. Antitumor study investigating the efficacy of SC134-TCB on implanted DMS79 tumor in NSG mice. DMS79 cells were implanted on day 1 and PBMCs dosed on days 8 and 22 at an E:T of 5:1. Gray arrows show biweekly administration of SC134-TCB (100 μg); upward ticks show atezolizumab dosing (200 μg). **C,** Tumor volume analysis, mixed-effects model *P* < 0.0001. Individual growth curves shown in Supplementary Fig. S10; **D,** OS analysis, log-rank Mantel–Cox *P* < 0.01. **E,** Phenotyping data assessing the percentage of live T cells (Tils) and (**F**) the percentage PD1/TIM3 double-positive Tils in terminal tumor samples, Mann–Whitney test, *P* = 0.0142. Antitumor study investigating the efficacy of reduced dose or reduced dose frequency of SC134-TCB on implanted DMS79 tumor in NSG mice. DMS79 cells were implanted on day 1 and PBMCs dosed on days 7 and 21 at an E:T of 5:1. SC134-TCB dosing either biweekly (six doses of 10 or 100 μg) or weekly (three doses of 100 μg). **G,** Tumor volume analysis, two-way ANOVA, *P* = 0.0004. Individual growth curves shown in Supplementary Fig. S11. **H,** OS, log-rank Mantel–Cox, *P* < 0.0001. Atez, atezolizumab.

There is ample evidence that the PDL1:PD1 checkpoint limits T-cell activity (22, 23). Several observations in this study prompted us to evaluate the impact of PDL1 inhibition in our NSG DMS79 tumor model. First, up to 50% PDL1-positive DMS79 cells were observed after 48-hour *ex vivo* SC134-TCB treatment in huPBMC cocultures (Fig. 5A). Additionally, *in vivo*, SC134-TCB treatment induced a significant increase in PDL1-positive DMS79 (Fig. 5B). Finally, Tils recovered at termination from SC134-TCB treatment groups displayed increased levels of exhaustion (Fig. 4D). Importantly, atezolizumab in combination with chemotherapy is now the standard of care for the treatment of ES-SCLC (24). Accordingly, it was important to evaluate whether atezolizumab-driven PDL1 inhibition could further improve the SC134-TCB antitumor activity. SC134-TCB, in the absence of PDL1 inhibition, exerted a significant tumor growth inhibition compared with the control group, confirming its impact across several donor effector cells (Fig. 5C; Supplementary Fig. S10). Biweekly administration of SC134-TCB led to a 66% (*P* < 0.0001) tumor growth inhibition compared with the tumor plus effector control group (Fig. 5C), resulting in 30% OS (*P* = 0.0054, Fig. 5D). Atezolizumab (200 μg, dosed biweekly) resulted in an initial slowing of the DMS79 growth in combination with the administered huPBMC, which recovered after day 25, resulting in a small but significant survival benefit (Fig. 5C and D). Notably, the addition of atezolizumab to the SC134-TCB regimen further improved the OS to 50%, a significant enhancement compared with the atezolizumab treatment group (*P* = 0.0031). Terminal tumor analysis revealed a trend toward increased Til recovery in the SC134-TCB–treated and atezolizumab combination groups, not reaching significance (Fig. 5E). Til exhaustion levels based on PD1 and Tim3 coexpression, however, were significantly reduced in the atezolizumab combination group (Fig. 5F), suggesting that PDL1 blockade may have reduced terminal exhaustion in the Tils.

In order to further refine the *in vivo* SC134-TCB dose response, we next evaluated a lower dose (10 μg) biweekly administered as well as a weekly dosing of 100 μg (Fig. 5G; Supplementary Fig. S11). Reducing the dosing frequency to weekly dosing maintained significant tumor growth inhibition (Fig. 5G) but led to a reduction in OS to 30% compared with 70% for biweekly dosing (Fig. 5H). Maintaining the biweekly regimen, but lowering the dose to 10 μg, seemed to initially produce superior tumor control, but this did not reach significance, compared with 100 μg, with overall similar survival being 70% for both groups.

### Cellular assessment of safety

**Figure 6.**
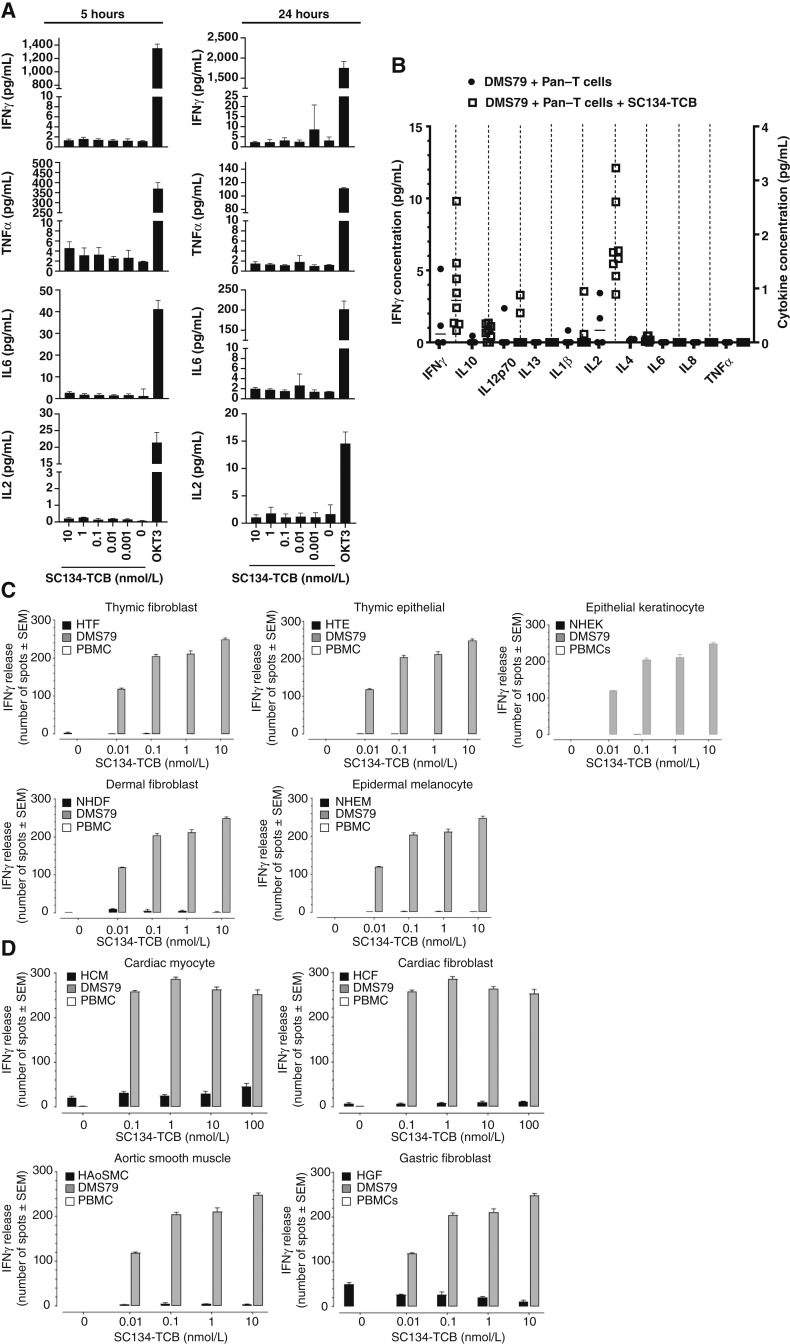
T cell–mediated cytokine production by SC134-TCB. **A,** Serum cytokine concentration after incubation of whole blood with SC134-TCB or huOKT3 for 5 and 24 hours from one representative donor of four analyzed. **B,** Cytokine detection in mouse sera 24 hours after one 100 μg SC134-TCB dose (DMS79 inoculation 8 days prior to pan-T and TCB dosing). Primary cell analysis of SC134-TCB–mediated T-cell activation. **C,** Reactivity measured by IFNγ ELISpot with normal primary human cells derived from the thymus and skin or (**D**) from cardiac and gastric tissues. DMS79 and PBMC only used as positive and negative controls, respectively. Representative data shown for two healthy PBMC donors.

Cytokine release syndrome (CRS) is a potentially life-threatening complication that can develop following the administration of an immunotherapy agent, causing a rapid release of proinflammatory cytokines into the blood from activated immune cells. The ability of SC134-TCB to induce the release of proinflammatory cytokines in the absence of target engagement was tested in whole blood from four healthy donors. Representative data for one donor are shown in Fig. 6A. SC134-TCB, over the concentration range tested, triggered minimal release (up to 24 hours) of IL2, IL6, TNFα, and IFNγ, all remaining below 10 pg/mL. In contrast, huOKT3 control showed robust cytokine production across all four cytokines. Cytokine production was also analyzed in mice sera, 24 hours after pan–T adoptive transfer and the first SC134-TCB dose, in an antitumor study (Fig. 6B). A small SC134-TCB–induced increase in IFNγ and IL2 was detectable, potentially already reflecting tumor target–mediated cytokine production. The other cytokines remained near the limit of detection.

To predict any potential off-tumor toxicity in the clinic, the activity of SC134-TCB was measured in the presence of normal primary cells derived from different human tissues, including two tissues where low amounts of FucGM1 were detected: thymus (3/3) and skin (2/3; pituitary cells were not available; Fig. 1J). Responses to thymic epithelial cells and thymic fibroblasts were very low (<10 spots) at all SC134-TCB concentrations tested, and the same was true for epithelial keratinocytes, dermal fibroblasts, and epidermal melanocytes with <20 spots on ELISpot (Fig. 6C). In contrast, SC134-TCB in combination with the target-positive DMS79 showed robust IFNγ induction. Next, we assessed the reactivity of SC134-TCB against a small panel of high-risk tissues, in which any reactivity to these cells could lead to fatal toxicity in the clinic. No responses were observed against cardiac myocytes, cardiac fibroblasts, aortic smooth muscle, and gastric fibroblasts at any SC134-TCB concentration (Fig. 6D).

## Discussion

Effective treatment options for patients with ES-SCLC remain limited despite the addition of PDL1 inhibitors to chemotherapy as a first-line strategy. Consequently, SCLC selective targets carry great value for the development of targeted therapies. The virtual absence of the FucGM1 glycolipid expression in normal healthy tissues, along with its low donor-dependent expression observed in the skin, pituitary, and thymus, combined with its extensive tumor expression, with more than 80% of positive patient-derived SCLC tissues, renders it an extremely attractive target for the development of a T cell–redirecting modality. Early-phase clinical results have already demonstrated that tumor selectivity of the target antigen is key to ensure a therapeutic window with T cell–engaging modalities (25).

Hybridoma-derived SC134 specifically targeted FucGM1 with no cross-reactivity to GM1, nor closely related gangliosides. SC134-TCB, based on h134, showed avid FucGM1 binding, due to its native antibody format, combined with nanomolar T-cell targeting, substantially weaker than the parental huOKT3 mAb, confirming the functional monovalency of the anti-huCD3 linkage (19). Coengagement of cellular FucGM1 and CD3 resulted in potent (subnanomolar to picomolar) *ex vivo* SCLC cell killing through the activation of pan–T cells or huPBMC from a range of donors and ensuing redirected cytotoxicity. SC134-TCB induced target-dependent activation and proliferation of both CD4 and CD8 T cells, coinciding with multifunctional cytokine production, encompassing IFNγ, IL2, and TNFα. Fc silencing of our full antibody-based format ensured that in the absence of target engagement, limited or no T-cell activation nor cytokine production was observed.

*In vivo*, using huPBMC-administered NSG mice, SC134-TCB treatment led to significant tumor growth inhibition, concomitant with a significant survival benefit. Previously, it has been postulated that CD3 affinity tuning in TCB formats aids biodistribution in preventing diversion of the TCB to the secondary lymphoid organs such as the spleen (26–28). Critically, SC134-TCB was robustly detected in tumors of treated mice, in contrast to the spleen where negligible TCB was detected, indicating effective tumor targeting. This was also evident from the shorter-duration antitumor study in which SC134-TCB induced a pronounced tumor T-cell infiltration, displaying an activated, effector phenotype. Although SC134-TCB induced 40% OS, terminal tumor analysis revealed increased levels of PD1/Tim3 double-positive Tils, markers previously identified in chronic viral infection models and representing T-cell exhaustion (29, 30). PDL1 inhibition is now standard of care in combination with chemotherapy for ES-SCLC. Additionally, terminal sample analysis revealed TCB-induced effector cell PD1 expression, combined with increased PDL1 expression by the tumor cells, suggesting an adaptive immune resistance mechanism, also observed in other preclinical TCB studies (31–33), and prompting us to evaluate the impact of blocking PDL1 *in vivo*. Combining the PDL1 inhibitor atezolizumab with SC134-TCB led to a significant increased OS, compared with the control group, in addition to a significant reduction in PD1/Tim3 double-positive Tils, indicating that PDL1 blockade improved Til exhaustion and the overall outcome of the study. The more efficacious TCB antitumor activity in combination with blockade of the PD1/PDL1 axis has also been observed across a number of other solid tumor-targeting TCB such as for the B7H6 and carcinoembryonic antigen T-cell engagers (34–36). Finally, reduced SC134-TCB dosing maintained the antitumor impact, with seemingly stronger early tumor growth inhibition, indicating that the reduced TCB-mediated T-cell activation retains effective tumor killing. Notwithstanding, in order to maintain long-term tumor control, strategies to impart immune memory formation or the *in situ* generation of additional effector cells are warranted.

Historically, stand-alone solid tumor–targeting antibodies have had limited impact. BMS-986012 is a clinical-stage, non-fucosylated, FucGM1-targeting antibody currently trialed in combination with nivolumab for the treatment of ES-SCLC (37). Two additional SCLC targets in clinical development are delta-like ligand 3 (DLL3), an inhibitor of Notch 1 ligand and B7H3 (CD276), an immunoregulatory protein member of the B7 family. Targeted drug delivery (antibody–drug conjugate) is currently being evaluated for the latter target (38), although the increase and decrease in the DLL3-targeted antibody–drug conjugate rovalpituzumab tesirine serve as a reminder that clinical success is not guaranteed. More recently, a B7H3-targeting TCB has been developed, equally comprising a “2 + 2” format, but with the anti-CD3 arm fused to the C-terminus of the antibody heavy chain (39). DLL3, on the other hand, has been at the forefront of many targeted therapies for SCLC, largely due to its tumor specificity, in which its cytoplasmic and cell surface expression contrast with its largely intracellular and restricted normal tissue expression (40, 41) Targeting DLL3, however, may pose a subtype-specific vulnerability as its expression may be preferentially associated with the SCLC A subtype because it is a direct transcriptional target of *ASCL1* (42). Nevertheless, a plethora of DLL3-targeting TCB are in clinical development, notably AMG757 (tarlatamab; ref. 43), BI 764532 (31), and HPN238 (44). Response rates from DeLLphi-301, in which tarlatamab was evaluated in patients with ES-SCLC that had progressed on therapy, were significantly better than those from standard care, suggesting that SCLC is a setting in which T-cell engaging modalities can offer benefit (45). However, toxicities, such as CRS, were evident in about half of patients, combined with less frequent, immune effector cell–associated neurotoxicity syndrome. CRS is commonly observed with TCB both in solid and liquid tumors and usually effectively managed with supportive care (46). Nevertheless, whole-blood assays in which SC134-TCB was incubated for up to 24 hours with whole blood from four healthy donors presented little evidence for CRS, likely a result from rendering the Fc portion of our format inert (19) combined with the absence of target. The potential for *off-tumor* toxicity was evaluated using primary cells derived from low-level FucGM1-expressing tissues (detected via IHC) as well as from critical organs, in combination with SC134-TCB and PBMCs. The absence of significant reactivity to the thymus and skin where low-level FucGM1 expression had been detected, in combination with the absence of reactivity to the small panel of high-risk tissues, suggests that SC134-TCB may not carry inherent toxicity liabilities, but a more extensive analysis is warranted.

In hematologic tumors, T-cell bispecific modalities have proven their merit, with several recent approvals (47). Solid tumors, however, are a more challenging setting, not least due to the need for tumor-specific targets to ensure a reasonable therapeutic window, in spite of TCB not relying on MHC expression for T cell–mediated target killing (48). Whether TCB impact is driven by the baseline T-cell infiltrate or whether external T-cell recruitment is necessary and sufficient for antitumor activity seems to be governed by a number of factors, such as the amount and quality of the resident Tils, the TCB target density and homogeneity, and the TCB dose (49, 50). Nonetheless, as higher levels of effectors are instrumental to better outcomes, strategies to increase their frequency, for instance, through tumor-specific vaccination and/or tumor vasculature targeting are actively being explored (39, 51). Additionally, combination strategies to further enhance effector T-cell expansion such as through CD28-driven or 4-1BB–driven costimulation, modulation of the regulatory T-cell compartment via anti-CTLA4, or checkpoint inhibition through PD1/PDL1 blockade have all preclinically demonstrated further improvements in antitumor effects of TCB (50). Validation of these TCB combination strategies in the clinic is eagerly awaited.

SC134-TCB, targeting a supremely SCLC-selective glycolipid, FucGM1, represents an attractive development candidate as an additional T cell–redirecting therapy option for patients with SCLC.

## Supplementary Material

Supplemental Table 1 EC50 values for cell binding

Supplemental Figure 1 Glycolipid binding validation and IHC

Supplemental Figure 2 Target-dependent SCLC binding

Supplemental Figure 3 Target-dependent killing

Supplemental Figure 4 Maximum cytotoxicity

Supplemental Figure 5 SC134-TCB induced IFNy

Supplemental Figure 6 Target-dependent multifunctional cytokine production

Supplemental Figure 7 Serum levels of SC134-TCB in NSG mice

Supplemental Figure 8 Tumour growth in the huPBMC admixed anti-tumour study

Supplemental Figure 9 Further in vivo validation: growth curves, IHC

Supplemental Figure 10 Individual tumour growth curves Atezolizumab study

Supplemental Figure 11 Individual tumour growth curves, dosing regimen study

Supplementary Method Protein sequence and generation of SC134-TCB

## References

[1] Dingemans A-MC , Früh M, Ardizzoni A, Besse B, Faivre-Finn C, Hendriks LE, . Small-cell lung cancer: ESMO Clinical Practice Guidelines for diagnosis, treatment and follow-up^☆^. Ann Oncol 2021;32:839–53. 33864941 10.1016/j.annonc.2021.03.207 PMC9464246

[2] Moreau P. , Garfall AL, van de Donk NWCJ, Nahi H, San-Miguel JF, Oriol A, . Teclistamab in relapsed or refractory multiple myeloma. N Engl J Med 2022;387:495–505. 35661166 10.1056/NEJMoa2203478 PMC10587778

[3] Chari A , Minnema MC, Berdeja JG, Oriol A, van de Donk NWCJ, Rodríguez-Otero P, . Talquetamab, a T-cell-redirecting GPRC5D bispecific antibody for multiple myeloma. N Engl J Med 2022;387:2232–44. 36507686 10.1056/NEJMoa2204591

[4] Feizi T . Demonstration by monoclonal antibodies that carbohydrate structures of glycoproteins and glycolipids are onco-developmental antigens. Nature 1985;314:53–7. 2579340 10.1038/314053a0

[5] Drivsholm L , Vangsted A, Pallesen T, Hansen M, Dombernowsky P, Hirsch F, . Fucosyl-GM1 in small-cell lung cancer. A comparison with the tumour marker neuron-specific enolase. Ann Oncol 1994;5:623–6. 7993838 10.1093/oxfordjournals.annonc.a058934

[6] Brezicka FT , Olling S, Nilsson O, Bergh J, Holmgren J, Sörenson S, . Immunohistological detection of fucosyl-GM1 ganglioside in human lung cancer and normal tissues with monoclonal antibodies. Cancer Res 1989;49:1300–5. 2645049

[7] Nilsson O. , Brezicka FT, Holmgren J, Sörenson S, Svennerholm L, Yngvason F, . Detection of a ganglioside antigen associated with small cell lung carcinomas using monoclonal antibodies directed against fucosyl-GM1. Cancer Res 1986;46:1403–7. 3002616

[8] Nilsson O , MÅnsson J-E, Brezicka T, Holmgren J, Lindholm L, Sörenson S, . Fucosyl-GM1 — a ganglioside associated with small cell lung carcinomas. Glycoconjugate J 1984;1:43–9.

[9] Brezicka FT , Olling S, Bergman B, Berggren H, Engström CP, Hammarström S, . Coexpression of ganglioside antigen Fuc-GM1, neural-cell adhesion molecule, carcinoembryonic antigen, and carbohydrate tumor-associated antigen CA 50 in lung cancer. Tumour Biol 1992;13:308–15. 1337798 10.1159/000217780

[10] Brezicka FT , Olling S, Bergman B, Berggren H, Engström CP, Holmgren J, . Immunohistochemical detection of two small cell lung carcinoma-associated antigens defined by MAbs F12 and 123C3 in bronchoscopy biopsy tissues. APMIS 1991;99:797–802. 1654059 10.1111/j.1699-0463.1991.tb01262.x

[11] Brezicka T , Bergman B, Olling S, Fredman P. Reactivity of monoclonal antibodies with ganglioside antigens in human small cell lung cancer tissues. Lung Cancer 2000;28:29–36. 10704706 10.1016/s0169-5002(99)00107-5

[12] Fredman P , Brezicka T, Holmgren J, Lindholm L, Nilsson O, Svennerholm L. Binding specificity of monoclonal antibodies to ganglioside, Fuc-GM1. Biochim Biophys Acta 1986;875:316–23. 3942769 10.1016/0005-2760(86)90182-7

[13] Kartal Yandım M , Apohan E, Baran Y. Therapeutic potential of targeting ceramide/glucosylceramide pathway in cancer. Cancer Chemother Pharmacol 2013;71:13–20. 23073611 10.1007/s00280-012-1984-x

[14] Tokuda N , Zhang Q, Yoshida S, Kusunoki S, Urano T, Furukawa K, . Genetic mechanisms for the synthesis of fucosyl GM1 in small cell lung cancer cell lines. Glycobiology 2006;16:916–25. 16880505 10.1093/glycob/cwl022

[15] Martín-Satué M , Marrugat R, Cancelas JA, Blanco J. Enhanced expression of alpha(1,3)-fucosyltransferase genes correlates with E-selectin-mediated adhesion and metastatic potential of human lung adenocarcinoma cells. Cancer Res 1998;58:1544–50. 9537262

[16] Vangsted A , Drivsholm L, Andersen E, Pallesen T, Zeuthen J, Wallin H. New serum markers for small-cell lung cancer. I. The ganglioside fucosyl-GM1. Cancer Detect Prev 1994;18:221–9. 8076384

[17] Vangsted AJ , Clausen H, Kjeldsen TB, White T, Sweeney B, Hakomori S, . Immunochemical detection of a small cell lung cancer-associated ganglioside (FucGM1) antigen in serum. Cancer Res 1991;51:2879–84. 1851663

[18] Adair JR , Athwal DS, Bodmer MW, Bright SM, Collins AM, Pulito VL, . Humanization of the murine anti-human CD3 monoclonal antibody OKT3. Hum Antibodies Hybridomas 1994;5:41–7. 7858182

[19] Santich BH , Park JA, Tran H, Guo H-F, Huse M, Cheung N-KV. Interdomain spacing and spatial configuration drive the potency of IgG-[L]-scFv T cell bispecific antibodies. Sci Transl Med 2020;12:eaax1315. 32161106 10.1126/scitranslmed.aax1315 PMC7437947

[20] Orcutt KD , Ackerman ME, Cieslewicz M, Quiroz E, Slusarczyk AL, Frangioni JV, . A modular IgG-scFv bispecific antibody topology. Protein Eng Des Sel 2010;23:221–8. 20019028 10.1093/protein/gzp077 PMC2841541

[21] Wilkinson I , Anderson S, Fry J, Julien LA, Neville D, Qureshi O, . Fc-engineered antibodies with immune effector functions completely abolished. PLoS One 2021;16:e0260954. 34932587 10.1371/journal.pone.0260954 PMC8691596

[22] Patsoukis N. , Wang Q, Strauss L, Boussiotis VA. Revisiting the PD-1 pathway. Sci Adv 2020;6:eabd2712. 32948597 10.1126/sciadv.abd2712 PMC7500922

[23] Budimir N , Thomas GD, Dolina JS, Salek-Ardakani S. Reversing T-cell exhaustion in cancer: Lessons learned from PD-1/PD-L1 immune checkpoint blockade. Cancer Immunol Res 2022;10:146–53. 34937730 10.1158/2326-6066.CIR-21-0515

[24] Horn L , Mansfield AS, Szczęsna A, Havel L, Krzakowski M, Hochmair MJ, . First-line Atezolizumab plus chemotherapy in extensive-stage small-cell lung cancer. N Engl J Med 2018;379:2220–9. 30280641 10.1056/NEJMoa1809064

[25] Kebenko M , Goebeler M-E, Wolf M, Hasenburg A, Seggewiss-Bernhardt R, Ritter B, . A multicenter phase 1 study of solitomab (MT110, AMG 110), a bispecific EpCAM/CD3 T-cell engager (BiTE^®^) antibody construct, in patients with refractory solid tumors. Oncoimmunology 2018;7:e1450710. 30221040 10.1080/2162402X.2018.1450710 PMC6136859

[26] Haber L , Olson K, Kelly MP, Crawford A, DiLillo DJ, Tavaré R, . Generation of T-cell-redirecting bispecific antibodies with differentiated profiles of cytokine release and biodistribution by CD3 affinity tuning. Sci Rep 2021;11:14397. 34257348 10.1038/s41598-021-93842-0 PMC8277787

[27] Mandikian D , Takahashi N, Lo AA, Li J, Eastham-Anderson J, Slaga D, . Relative target affinities of T-cell-dependent bispecific antibodies determine biodistribution in a solid tumor mouse model. Mol Cancer Ther 2018;17:776–85. 29339550 10.1158/1535-7163.MCT-17-0657

[28] Trinklein ND , Pham D, Schellenberger U, Buelow B, Boudreau A, Choudhry P, . Efficient tumor killing and minimal cytokine release with novel T-cell agonist bispecific antibodies. MAbs 2019;11:639–52. 30698484 10.1080/19420862.2019.1574521 PMC6601548

[29] Zhou Q , Munger ME, Veenstra RG, Weigel BJ, Hirashima M, Munn DH, . Coexpression of Tim-3 and PD-1 identifies a CD8+ T-cell exhaustion phenotype in mice with disseminated acute myelogenous leukemia. Blood 2011;117:4501–10. 21385853 10.1182/blood-2010-10-310425 PMC3099570

[30] Jin HT , Anderson AC, Tan WG, West EE, Ha S-J, Araki K, . Cooperation of Tim-3 and PD-1 in CD8 T-cell exhaustion during chronic viral infection. Proc Natl Acad Sci U S A 2010;107:14733–8. 20679213 10.1073/pnas.1009731107 PMC2930455

[31] Hipp S , Voynov V, Drobits-Handl B, Giragossian C, Trapani F, Nixon AE, . A bispecific DLL3/CD3 IgG-like T-cell engaging antibody induces antitumor responses in small cell lung cancer. Clin Cancer Res 2020;26:5258–68. 32554516 10.1158/1078-0432.CCR-20-0926

[32] Junttila TT , Li J, Johnston J, Hristopoulos M, Clark R, Ellerman D, . Antitumor efficacy of a bispecific antibody that targets HER2 and activates T cells. Cancer Res 2014;74:5561–71. 25228655 10.1158/0008-5472.CAN-13-3622-T

[33] Bacac M , Fauti T, Sam J, Colombetti S, Weinzierl T, Ouaret D, . A novel carcinoembryonic antigen T-cell bispecific antibody (CEA TCB) for the treatment of solid tumors. Clin Cancer Res 2016;22:3286–97. 26861458 10.1158/1078-0432.CCR-15-1696

[34] Zhang W , Auguste A, Liao X, Walterskirchen C, Bauer K, Lin Y-H, . A novel B7-H6-targeted IgG-like T cell-engaging antibody for the treatment of gastrointestinal tumors. Clin Cancer Res 2022;28:5190–201. 36166004 10.1158/1078-0432.CCR-22-2108 PMC9713360

[35] Sam J , Colombetti S, Fauti T, Roller A, Biehl M, Fahrni L, . Combination of T-cell bispecific antibodies with PD-L1 checkpoint inhibition elicits superior anti-tumor activity. Front Oncol 2020;10:575737. 33330050 10.3389/fonc.2020.575737 PMC7735156

[36] Wang N , Patel H, Schneider IC, Kai X, Varshney AK, Zhou L. An optimal antitumor response by a novel CEA/CD3 bispecific antibody for colorectal cancers. Antibody Ther 2021;4:90–100. 10.1093/abt/tbab009 PMC8220303 34169228

[37] Chu Q , Leighl NB, Surmont V, van Herpen C, Sibille A, Markman B, . BMS-986012, an anti-fucosyl-GM1 monoclonal antibody as monotherapy or in combination with Nivolumab in relapsed/refractory SCLC: results from a first-in-human phase 1/2 study. JTO Clin Res Rep 2022;3:100400. 36275912 10.1016/j.jtocrr.2022.100400 PMC9579497

[38] Yamato M , Hasegawa J, Maejima T, Hattori C, Kumagai K, Watanabe A, . DS-7300a, a DNA topoisomerase I inhibitor, DXd-based antibody-drug conjugate targeting B7-H3, exerts potent antitumor activities in preclinical models. Mol Cancer Ther 2022;21:635–46. 35149548 10.1158/1535-7163.MCT-21-0554 PMC9377751

[39] Zekri L , Lutz M, Prakash N, Manz T, Klimovich B, Mueller S, . An optimized IgG-based B7-H3xCD3 bispecific antibody for treatment of gastrointestinal cancers. Mol Ther 2023;31:1033–45. 36793213 10.1016/j.ymthe.2023.02.010 PMC10124076

[40] Rudin CM , Reck M, Johnson ML, Blackhall F, Hann CL, Yang JC-H, . Emerging therapies targeting the delta-like ligand 3 (DLL3) in small cell lung cancer. J Hematol Oncol 2023;16:66. 37355629 10.1186/s13045-023-01464-y PMC10290806

[41] Chapman G , Sparrow DB, Kremmer E, Dunwoodie SL. Notch inhibition by the ligand DELTA-LIKE 3 defines the mechanism of abnormal vertebral segmentation in spondylocostal dysostosis. Hum Mol Genet 2011;20:905–16. 21147753 10.1093/hmg/ddq529

[42] Rudin CM , Poirier JT, Byers LA, Dive C, Dowlati A, George J, . Molecular subtypes of small cell lung cancer: a synthesis of human and mouse model data. Nat Rev Cancer 2019;19:289–97. 30926931 10.1038/s41568-019-0133-9 PMC6538259

[43] Giffin MJ , Cooke K, Lobenhofer EK, Estrada J, Zhan J, Deegen P, . AMG 757, a half-life extended, DLL3-targeted bispecific T-cell engager, shows high potency and sensitivity in preclinical models of small-cell lung cancer. Clin Cancer Res 2021;27:1526–37. 33203642 10.1158/1078-0432.CCR-20-2845

[44] Johnson ML , Dy GK, Mamdani H, Dowlati A, Schoenfeld AJ, Pacheco JM, . Interim results of an ongoing phase 1/2a study of HPN328, a tri-specific, half-life extended, DLL3-targeting, T-cell engager, in patients with small cell lung cancer and other neuroendocrine cancers. J Clin Oncol 2022;40(Suppl 16):8566.

[45] Ahn M-J , Cho BC, Felip E, Korantzis I, Ohashi K, Majem M, . Tarlatamab for patients with previously treated small-cell lung cancer. N Engl J Med 2023;389:2063–75. 37861218 10.1056/NEJMoa2307980

[46] Van De Vyver AJ , Marrer-Berger E, Wang K, Lehr T, Walz AC. Cytokine release syndrome by T-cell–redirecting therapies: can we predict and modulate patient risk? Clin Cancer Res 2021;27:6083–94. 34162679 10.1158/1078-0432.CCR-21-0470

[47] Tapia-Galisteo A , Álvarez-Vallina L, Sanz L. Bi- and trispecific immune cell engagers for immunotherapy of hematological malignancies. J Hematol Oncol 2023;16:83. 37501154 10.1186/s13045-023-01482-w PMC10373336

[48] Arvedson T. , Bailis JM, Britten CD, Klinger M, Nagorsen D, Coxon A, . Targeting solid tumors with bispecific T cell engager immune therapy. Annu Rev Cancer Biol 2022;6:17–34.

[49] You R , Artichoker J, Ray A, Gonzalez Velozo H, Rock DA, Conner KP, . Visualizing spatial and stoichiometric barriers to bispecific T-cell engager efficacy. Cancer Immunol Res 2022;10:698–712. 35413104 10.1158/2326-6066.CIR-21-0594 PMC9177795

[50] Belmontes B , Sawant DV, Zhong W, Tan H, Kaul A, Aeffner F, . Immunotherapy combinations overcome resistance to bispecific T cell engager treatment in T cell-cold solid tumors. Sci Transl Med 2021;13:eabd1524. 34433637 10.1126/scitranslmed.abd1524

[51] Middelburg J , Sluijter M, Schaap G, Göynük B, Lloyd K, Ovcinnikovs V, . T-cell stimulating vaccines empower CD3 bispecific antibody therapy in solid tumors. Nat Commun 2024;15:48. 38167722 10.1038/s41467-023-44308-6 PMC10761684

