# Supplementary material for: SC134-TCB Targeting Fucosyl-GM1, a T Cell–Engaging Antibody with Potent Antitumor Activity in Preclinical Small Cell Lung Cancer Models
Source: Mol Cancer Ther. 2024 Aug 26;23(11):1626–38. doi: 10.1158/1535-7163.MCT-24-0187 (PMC11532774; doi:10.1158/1535-7163.MCT-24-0187)
Supplement: Supplemental Table 1 — EC50 values for cell binding [file mct-24-0187_supplemental_table_1_suppst1.docx]

| **Constructs** | **Cell binding** | | | |
| --- | --- | --- | --- | --- |
|  | DMS79 | DMS153 | H740 | T cells |
| SC134-TCB | 2.51E-08 | 3.39E-08 | 1.78E-08 | 2.92E-08 |
| B12-TCB | N/A | N/A | N/A | 3.25E-08 |
| h134 | 0.97E-08 | 4.20E-08 | 1.36E-08 |  |
| huOKT3 | N/A | N/A | N/A | 3.34E-10 |

**Supplemental Table 1**. EC_50_ (mol/L) for SC134-TCB cell binding compared to parental and control mAbs
