# Supplementary material for: SC134-TCB Targeting Fucosyl-GM1, a T Cell–Engaging Antibody with Potent Antitumor Activity in Preclinical Small Cell Lung Cancer Models
Source: Mol Cancer Ther. 2024 Aug 26;23(11):1626–38. doi: 10.1158/1535-7163.MCT-24-0187 (PMC11532774; doi:10.1158/1535-7163.MCT-24-0187)
Supplement: Supplemental Figure 1 — Glycolipid binding validation and IHC [file mct-24-0187_supplemental_figure_1_suppsf1.pptx]

## Slide 1
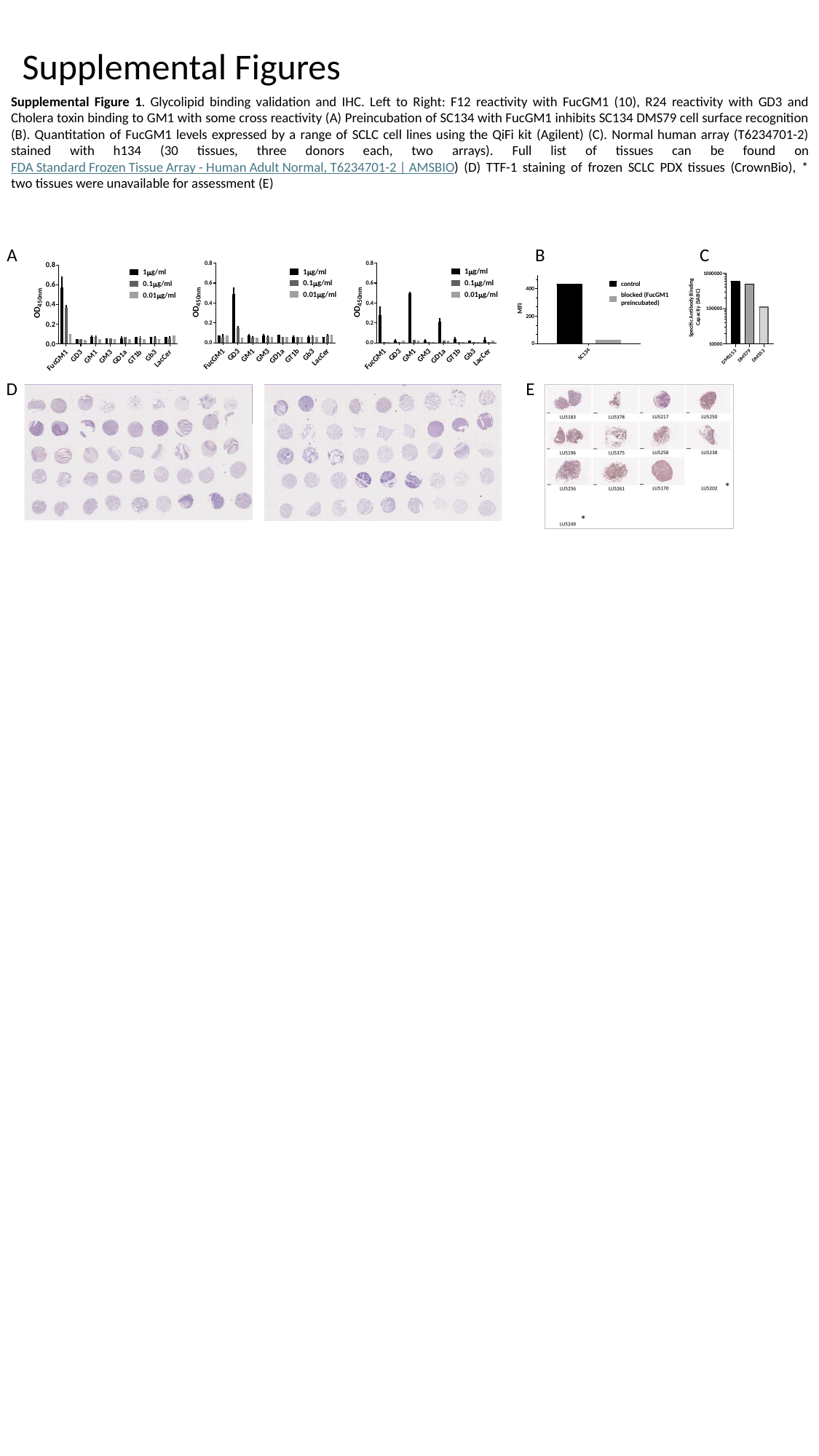

Supplemental Figures
Supplemental Figure 1. Glycolipid binding validation and IHC. Left to Right: F12 reactivity with FucGM1 (10), R24 reactivity with GD3 and Cholera toxin binding to GM1 with some cross reactivity (A) Preincubation of SC134 with FucGM1 inhibits SC134 DMS79 cell surface recognition (B). Quantitation of FucGM1 levels expressed by a range of SCLC cell lines using the QiFi kit (Agilent) (C). Normal human array (T6234701-2) stained with h134 (30 tissues, three donors each, two arrays). Full list of tissues can be found on FDA Standard Frozen Tissue Array - Human Adult Normal, T6234701-2 | AMSBIO) (D) TTF-1 staining of frozen SCLC PDX tissues (CrownBio), * two tissues were unavailable for assessment (E)
A
B
C
D
E
*
*
