# Supplementary material for: SC134-TCB Targeting Fucosyl-GM1, a T Cell–Engaging Antibody with Potent Antitumor Activity in Preclinical Small Cell Lung Cancer Models
Source: Mol Cancer Ther. 2024 Aug 26;23(11):1626–38. doi: 10.1158/1535-7163.MCT-24-0187 (PMC11532774; doi:10.1158/1535-7163.MCT-24-0187)
Supplement: Supplemental Figure 2 — Target-dependent SCLC binding [file mct-24-0187_supplemental_figure_2_suppsf2.pptx]

## Slide 1
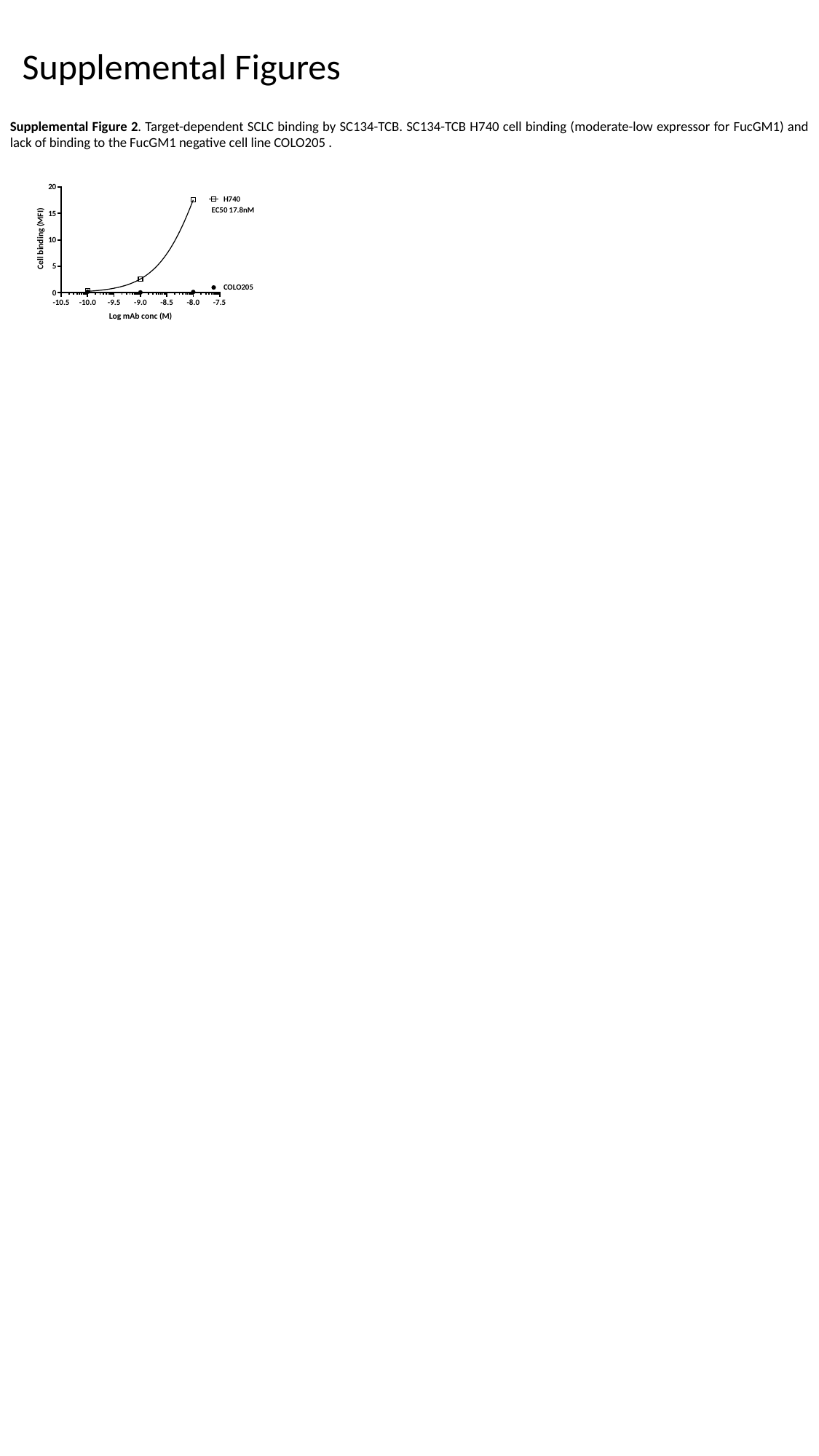

Supplemental Figures
Supplemental Figure 2. Target-dependent SCLC binding by SC134-TCB. SC134-TCB H740 cell binding (moderate-low expressor for FucGM1) and lack of binding to the FucGM1 negative cell line COLO205 .
