# Supplementary material for: SC134-TCB Targeting Fucosyl-GM1, a T Cell–Engaging Antibody with Potent Antitumor Activity in Preclinical Small Cell Lung Cancer Models
Source: Mol Cancer Ther. 2024 Aug 26;23(11):1626–38. doi: 10.1158/1535-7163.MCT-24-0187 (PMC11532774; doi:10.1158/1535-7163.MCT-24-0187)
Supplement: Supplemental Figure 3 — Target-dependent killing [file mct-24-0187_supplemental_figure_3_suppsf3.pptx]

## Slide 1
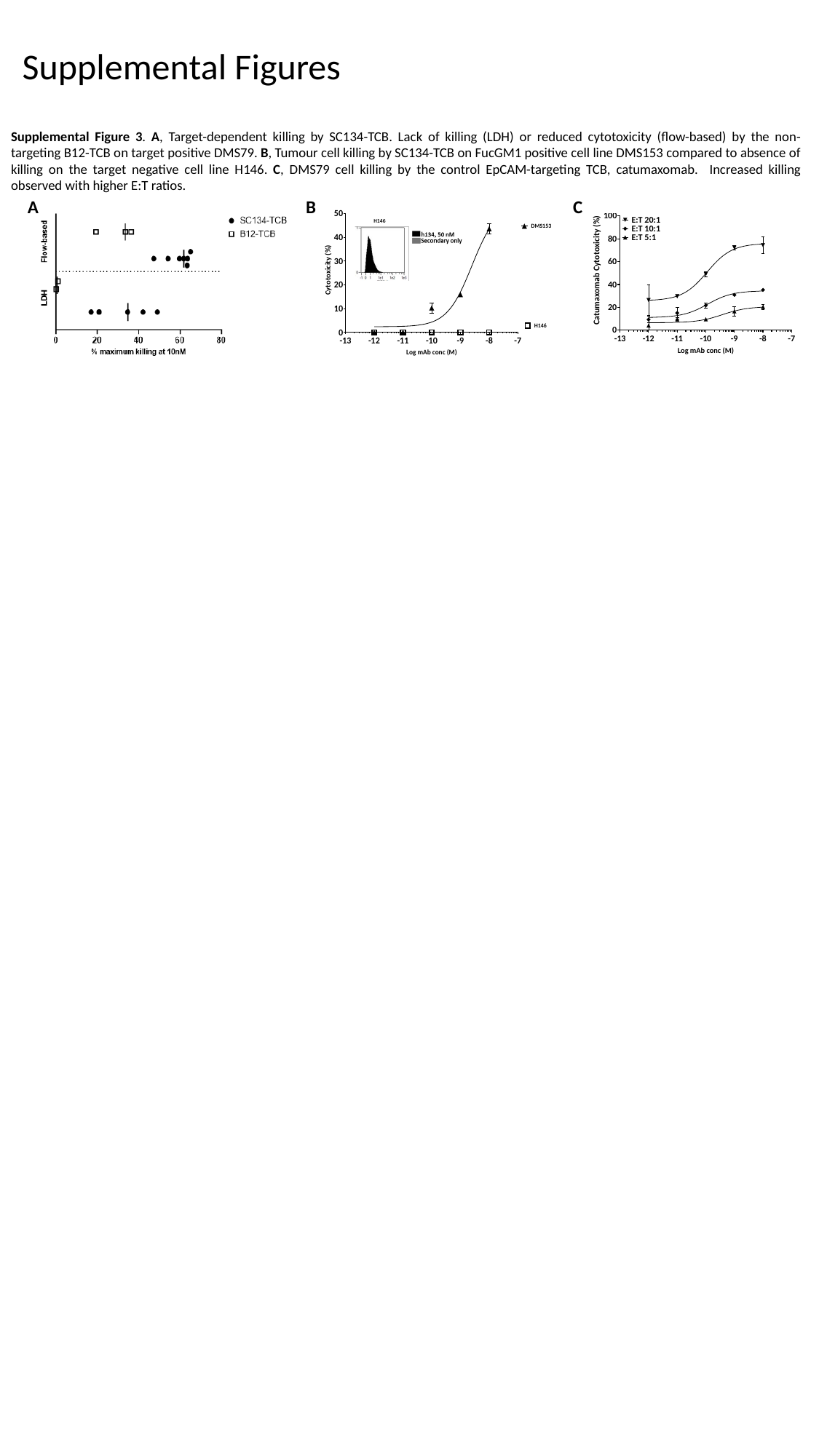

Supplemental Figures
Supplemental Figure 3. A, Target-dependent killing by SC134-TCB. Lack of killing (LDH) or reduced cytotoxicity (flow-based) by the non-targeting B12-TCB on target positive DMS79. B, Tumour cell killing by SC134-TCB on FucGM1 positive cell line DMS153 compared to absence of killing on the target negative cell line H146. C, DMS79 cell killing by the control EpCAM-targeting TCB, catumaxomab. Increased killing observed with higher E:T ratios.
A
C
B
