# Supplementary material for: SC134-TCB Targeting Fucosyl-GM1, a T Cell–Engaging Antibody with Potent Antitumor Activity in Preclinical Small Cell Lung Cancer Models
Source: Mol Cancer Ther. 2024 Aug 26;23(11):1626–38. doi: 10.1158/1535-7163.MCT-24-0187 (PMC11532774; doi:10.1158/1535-7163.MCT-24-0187)
Supplement: Supplemental Figure 4 — Maximum cytotoxicity [file mct-24-0187_supplemental_figure_4_suppsf4.pptx]

## Slide 1
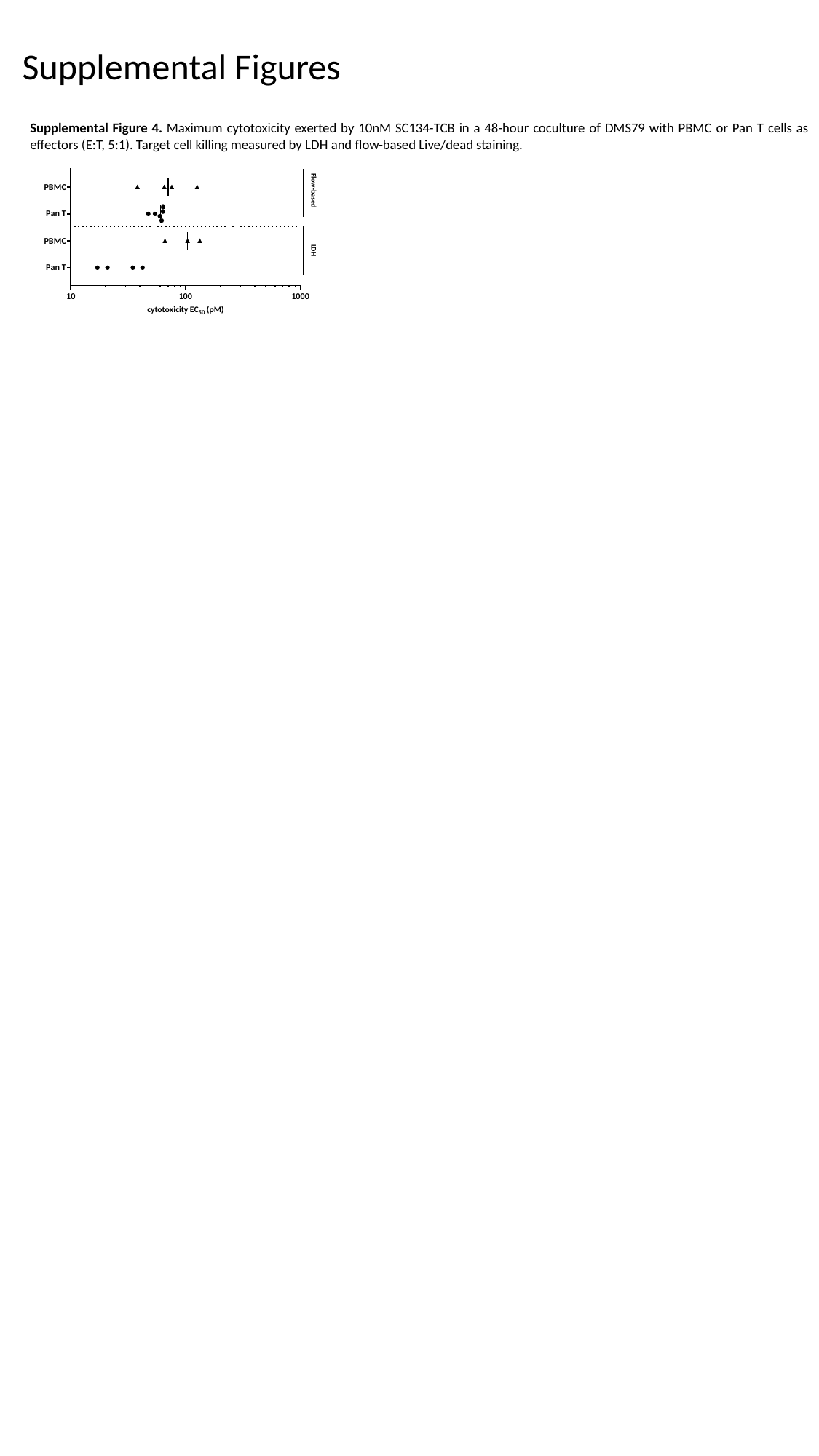

Supplemental Figures
Supplemental Figure 4. Maximum cytotoxicity exerted by 10nM SC134-TCB in a 48-hour coculture of DMS79 with PBMC or Pan T cells as effectors (E:T, 5:1). Target cell killing measured by LDH and flow-based Live/dead staining.
