# Supplementary material for: SC134-TCB Targeting Fucosyl-GM1, a T Cell–Engaging Antibody with Potent Antitumor Activity in Preclinical Small Cell Lung Cancer Models
Source: Mol Cancer Ther. 2024 Aug 26;23(11):1626–38. doi: 10.1158/1535-7163.MCT-24-0187 (PMC11532774; doi:10.1158/1535-7163.MCT-24-0187)
Supplement: Supplemental Figure 5 — SC134-TCB induced IFNy [file mct-24-0187_supplemental_figure_5_suppsf5.pptx]

## Slide 1
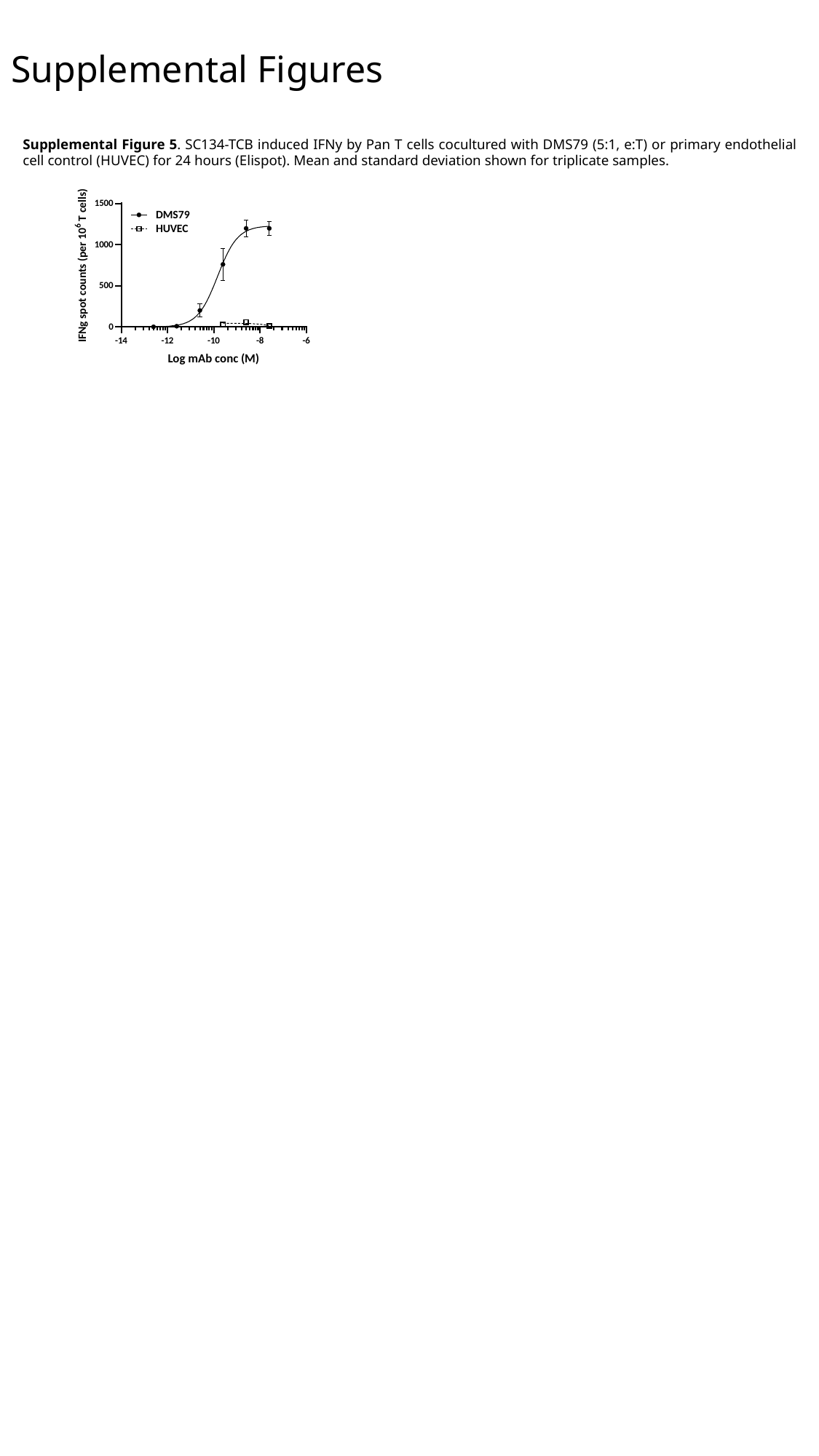

Supplemental Figures
Supplemental Figure 5. SC134-TCB induced IFNy by Pan T cells cocultured with DMS79 (5:1, e:T) or primary endothelial cell control (HUVEC) for 24 hours (Elispot). Mean and standard deviation shown for triplicate samples.
