# Supplementary material for: SC134-TCB Targeting Fucosyl-GM1, a T Cell–Engaging Antibody with Potent Antitumor Activity in Preclinical Small Cell Lung Cancer Models
Source: Mol Cancer Ther. 2024 Aug 26;23(11):1626–38. doi: 10.1158/1535-7163.MCT-24-0187 (PMC11532774; doi:10.1158/1535-7163.MCT-24-0187)
Supplement: Supplemental Figure 6 — Target-dependent multifunctional cytokine production [file mct-24-0187_supplemental_figure_6_suppsf6.pptx]

## Slide 1
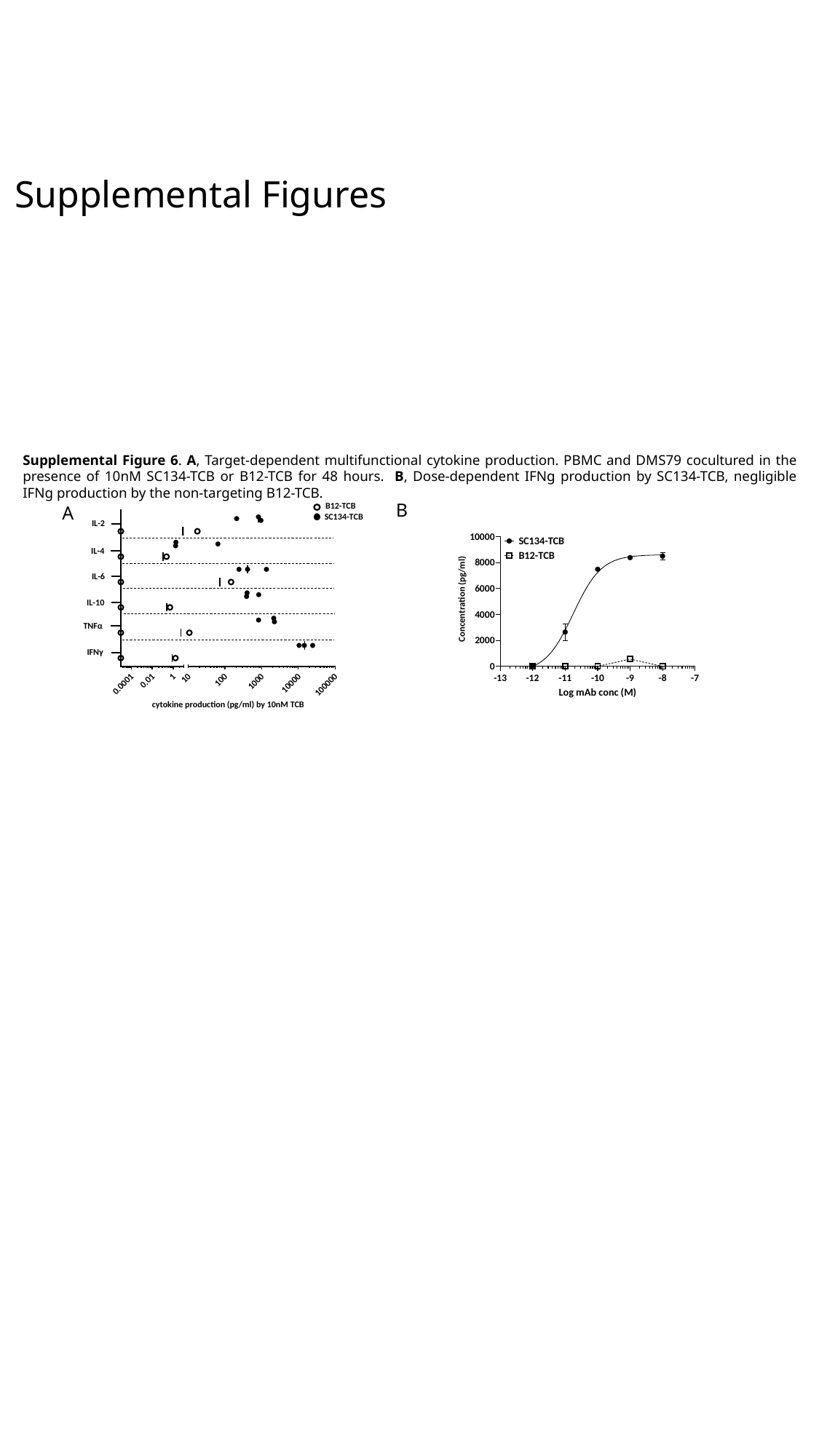

Supplemental Figures
Supplemental Figure 6. A, Target-dependent multifunctional cytokine production. PBMC and DMS79 cocultured in the presence of 10nM SC134-TCB or B12-TCB for 48 hours. B, Dose-dependent IFNg production by SC134-TCB, negligible IFNg production by the non-targeting B12-TCB.
B
A
