# Supplementary material for: SC134-TCB Targeting Fucosyl-GM1, a T Cell–Engaging Antibody with Potent Antitumor Activity in Preclinical Small Cell Lung Cancer Models
Source: Mol Cancer Ther. 2024 Aug 26;23(11):1626–38. doi: 10.1158/1535-7163.MCT-24-0187 (PMC11532774; doi:10.1158/1535-7163.MCT-24-0187)
Supplement: Supplemental Figure 8 — Tumour growth in the huPBMC admixed anti-tumour study [file mct-24-0187_supplemental_figure_8_suppsf8.pptx]

## Slide 1
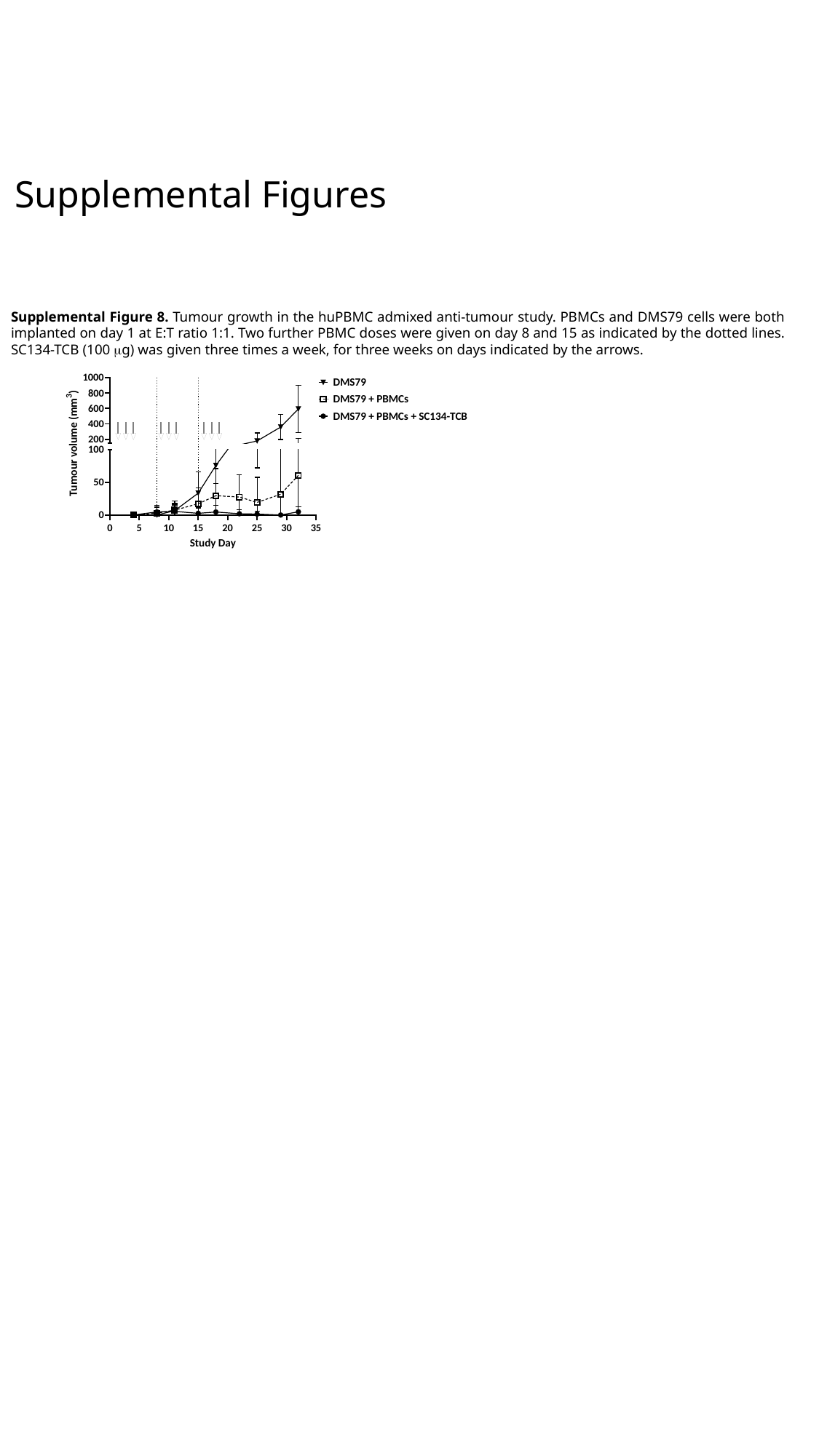

Supplemental Figures
Supplemental Figure 8. Tumour growth in the huPBMC admixed anti-tumour study. PBMCs and DMS79 cells were both implanted on day 1 at E:T ratio 1:1. Two further PBMC doses were given on day 8 and 15 as indicated by the dotted lines. SC134-TCB (100 μg) was given three times a week, for three weeks on days indicated by the arrows.
