# Supplementary material for: SC134-TCB Targeting Fucosyl-GM1, a T Cell–Engaging Antibody with Potent Antitumor Activity in Preclinical Small Cell Lung Cancer Models
Source: Mol Cancer Ther. 2024 Aug 26;23(11):1626–38. doi: 10.1158/1535-7163.MCT-24-0187 (PMC11532774; doi:10.1158/1535-7163.MCT-24-0187)
Supplement: Supplemental Figure 9 — Further in vivo validation: growth curves, IHC [file mct-24-0187_supplemental_figure_9_suppsf9.pptx]

## Slide 1
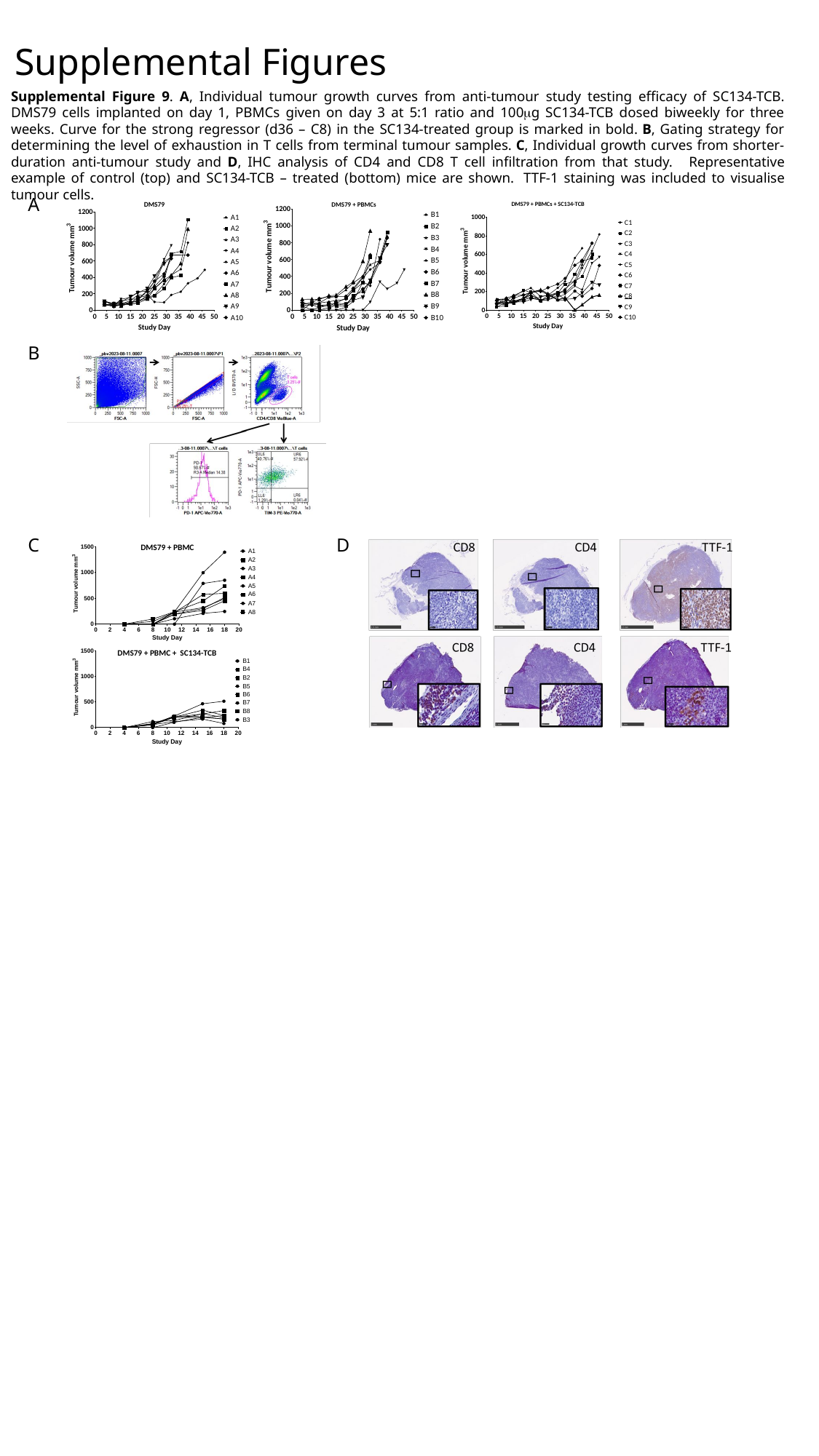

Supplemental Figures
Supplemental Figure 9. A, Individual tumour growth curves from anti-tumour study testing efficacy of SC134-TCB. DMS79 cells implanted on day 1, PBMCs given on day 3 at 5:1 ratio and 100μg SC134-TCB dosed biweekly for three weeks. Curve for the strong regressor (d36 – C8) in the SC134-treated group is marked in bold. B, Gating strategy for determining the level of exhaustion in T cells from terminal tumour samples. C, Individual growth curves from shorter-duration anti-tumour study and D, IHC analysis of CD4 and CD8 T cell infiltration from that study.  Representative example of control (top) and SC134-TCB – treated (bottom) mice are shown.  TTF-1 staining was included to visualise tumour cells.
A
B
C
D
