# Supplementary material for: SC134-TCB Targeting Fucosyl-GM1, a T Cell–Engaging Antibody with Potent Antitumor Activity in Preclinical Small Cell Lung Cancer Models
Source: Mol Cancer Ther. 2024 Aug 26;23(11):1626–38. doi: 10.1158/1535-7163.MCT-24-0187 (PMC11532774; doi:10.1158/1535-7163.MCT-24-0187)
Supplement: Supplemental Figure 10 — Individual tumour growth curves Atezolizumab study [file mct-24-0187_supplemental_figure_10_suppsf10.pptx]

## Slide 1
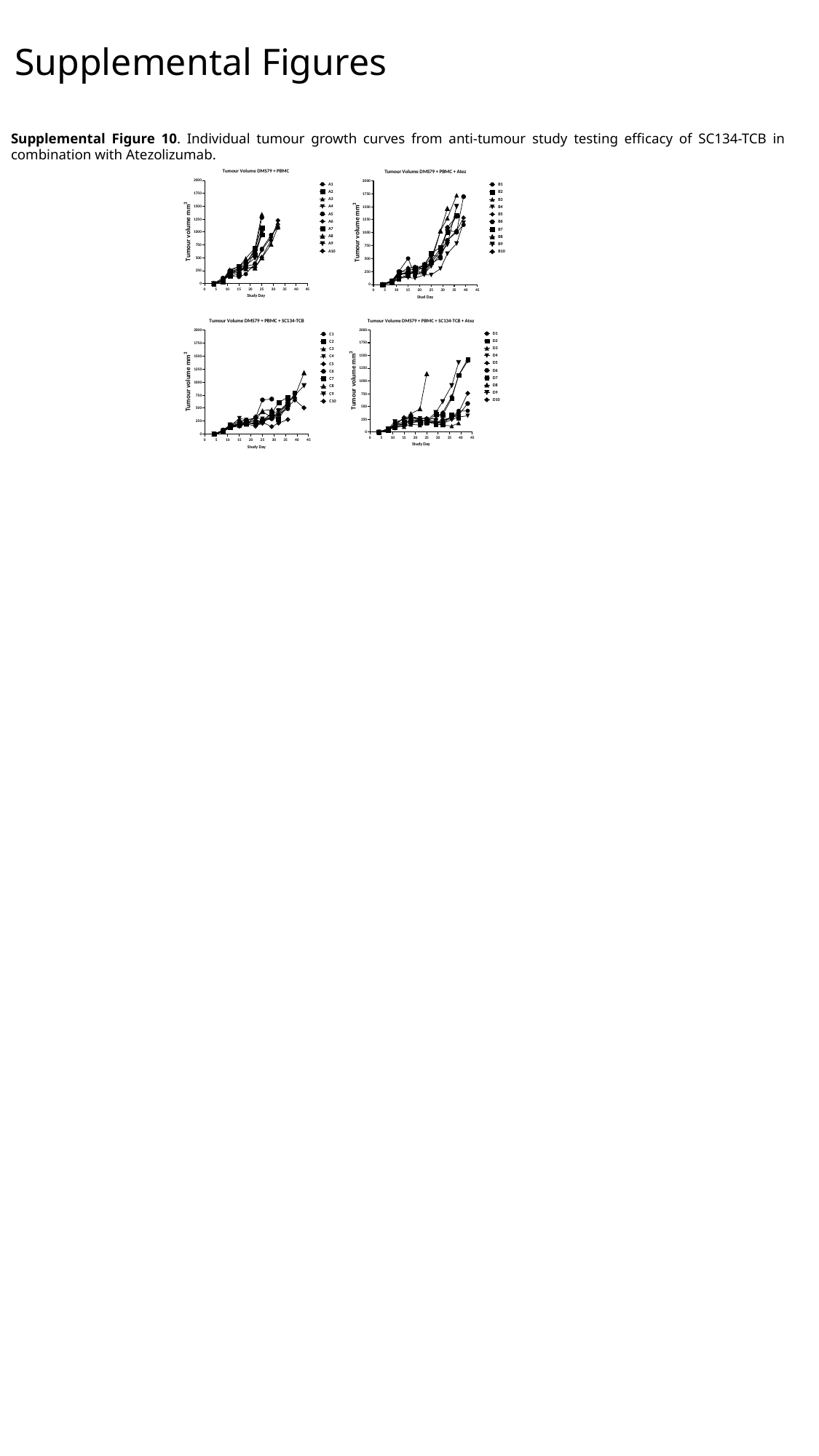

Supplemental Figures
Supplemental Figure 10. Individual tumour growth curves from anti-tumour study testing efficacy of SC134-TCB in combination with Atezolizumab.
