# Supplementary material for: SC134-TCB Targeting Fucosyl-GM1, a T Cell–Engaging Antibody with Potent Antitumor Activity in Preclinical Small Cell Lung Cancer Models
Source: Mol Cancer Ther. 2024 Aug 26;23(11):1626–38. doi: 10.1158/1535-7163.MCT-24-0187 (PMC11532774; doi:10.1158/1535-7163.MCT-24-0187)
Supplement: Supplementary Method — Protein sequence and generation of SC134-TCB [file mct-24-0187_supplementary_method_suppsm.docx]

**Supplemental Data**

#### Heavy Chain Protein Sequence of SC134-TCB

AVQLQESGPGLVKPSQTLSLTCTVTGYSITSGYSWHWIRQAPGKGLEWVGYIHYRGNTNYNPSLKSRVTISRDTSKNQFSLKLSSVTTEDTATYYCAQEPVRNYAMDFWGQGTLVTVSSASTKGPSVFPLAPSSKSTSGGTAALGCLVKDYFPEPVTVSWNSGALTSGVHTFPAVLQSSGLYSLSSVVTVPSSSLGTQTYICNVNHKPSNTKVDKKVEPKSCDKTHTCPPCPAPESTRGPSVFLFPPKPKDTLMISRTPEVTCVVVDVSHEDPEVKFNWYVDGVEVHNAKTKPREEQYNSTYRVVSVLTVLHQDWLNGKEYKCKVSNKALPAPIEKTISKAKGQPREPQVYTLPPSRDELTKNQVSLTCLVKGFYPSDIAVEWESNGQPENNYKTTPPVLDSDGSFFLYSKLTVDKSRWQQGNVFSCSVMHEALHNHYTQKSLSLSPGK

#### Light Chain protein sequence of SC134-TCB

DIVMTQSPATLSVSPGERATLSCRASQSISDDLHWYQQKPGKAPKLLIKYVSQSISGIPSRFSGSGSRSDFTLTISSLEPEDFALYYCQNGHGFPPTFGQGTKLEIKRTVAAPSVFIFPPSDEQLKSGTASVVCLLNNFYPREAKVQWKVDNALQSGNSQESVTEQDSKDSTYSLSSTLTLSKADYEKHKVYACEVTHQGLSSPVTKSFNRGECGGGGSGGGGSGGGGSQVQLVQSGGGVVQPGRSLRLSCKASGYTFTRYTMHWVRQAPGKCLEWIGYINPSRGYTNYNQKFKDRFTISRDNSKNTAFLQMDSLRPEDTGVYFCARYYDDHYSLDYWGQGTPVTVSSGGGGSGGGGSGGGGSGGGGSGGGGSGGGGSDIQMTQSPSSLSASVGDRVTITCSASSSVSYMNWYQQTPGKAPKRWIYDTSKLASGVPSRFSGSGSGTDYTFTISSLQPEDIATYYCQQWSSNPFTFGCGTKLITR

#### Generation of SC134-TCB

The DNA sequence of SC134-TCB was cloned into a proprietary vector system (Evitria AG) using conventional cloning techniques and correctness of the sequences verified with Sanger sequencing. Suspension-adapted CHO K1 cells (serum-free, animal component-free medium) were transfected for production and supernatant harvested by centrifugation and subsequent filtration. The antibody was purified using MabSelect™ SuRe™ Protein A affinity chromatography, monomericity assessed using SEC-HPLC and endotoxin content determined (<1EU/mg).
